# Murine xenograft bioreactors for human immunopeptidome discovery

**DOI:** 10.1038/s41598-019-54700-2

**Published:** 2019-12-06

**Authors:** James M. Heather, Paisley T. Myers, Feng Shi, Mohammad Ovais Aziz-Zanjani, Keira E. Mahoney, Matthew Perez, Benjamin Morin, Christine Brittsan, Jeffrey Shabanowitz, Donald F. Hunt, Mark Cobbold

**Affiliations:** 1 Center for Cancer Immunology, Massachusetts General Hospital, Boston, Massachusetts USA; 2 Department of Medicine, Harvard Medical School, Boston, Massachusetts USA; 3 Agenus Inc., Lexington, Massachusetts, USA; 4 Department of Chemistry, University of Virginia, Charlottesville, Virginia USA; 5 Department of Pathology, University of Virginia, Charlottesville, Virginia USA

**Keywords:** Immunology, Antigen processing and presentation, MHC class I

## Abstract

The study of peptides presented by MHC class I and class II molecules is limited by the need for relatively large cell numbers, especially when studying post-translationally modified or otherwise rare peptide species. To overcome this problem, we pose the hypothesis that human cells grown as xenografts in immunodeficient mice should produce equivalent immunopeptidomes as cultured cells. Comparing human cell lines grown either *in vitro* or as murine xenografts, we show that the immunopeptidome is substantially preserved. Numerous features are shared across both sample types, including peptides and proteins featured, length distributions, and HLA-binding motifs. Peptides well-represented in both groups were from more abundant proteins, or those with stronger predicted HLA binding affinities. Samples grown *in vivo* also recapitulated a similar phospho-immunopeptidome, with common sequences being those found at high copy number on the cell surface. These data indicate that xenografts are indeed a viable methodology for the production of cells for immunopeptidomic discovery.

## Introduction

The discovery and quantification of peptides presented to the immune system by MHC class I or class II molecules is a fundamental requirement for a full understanding of the adaptive immune system. The study of these peptides through mass spectrometry (MS) – termed immunopeptidomics, MHC ligandomics, or MHC peptidomics – has advanced much in recent years, with improvements in MS analysis, bioinformatics, and sample handling enabling the detection of in excess of 10,000 peptide sequences per sample in some experiments^1^, up from approximately ten per study at the inception of the field almost thirty years ago^2,3^.

Peptides bound by MHC (pMHC) make up only a small fraction of the cell mass and many peptide species are present at less than one molecule per cell, thus a major challenge for the field is the need for relatively large cell numbers or tissue volumes for initial sample input. Typical experiments often aim for 0.5–1 × 10^8^ cells^4^, depending on the particular setup of a given lab. This requirement becomes an even greater limitation when aiming to study specific, rarer peptide species presented by MHC, such as those bearing post-translational modifications (PTMs). These peptides occupy only a small fraction of the immunopeptidome and are correspondingly harder to reliably detect. For example, immunopeptidomics experiments studying enriched phosphorylated peptides – derived from the most common PTM in the cell^5^ – typically harvest in the range of 0.5–1 × 10^9^ cell equivalents to achieve sufficient material for detection^6^.

This requirement impacts immunopeptidomic experiments in several ways. For clinical samples, it means that only samples of sufficient size can be processed. This limits both the number and type of feasible samples, and reduces the amount of material available for further or parallel experiments. For cell lines and organoids, protracted *in vitro* culturing is required to generate sufficient cells for analysis. This can be slow, expensive, impractical (requiring both lots of operator time and incubator space), and could potentially introduce experimental bias and confounders.

We sought to address this problem by testing the hypothesis that cell lines grown as xenografts in immunodeficient mice should present equivalent peptide repertoires as those grown traditionally in culture, providing an alternate means to generate sufficient cell quantities.

Improvements in murine xenograft technology have been driven by extensive research in the fields of stem cell engraftment and patient-specific cancer treatment. Cells grown in immunodeficient mice (‘*in muridae*’) have been shown to recapitulate the gene expression profiles of those cultured *in vitro*^7^, particularly when only minimally passaged in mice^8^. More importantly, protein expression and even phosphorylation patterns are conserved^9^. Use of increasingly refined immunodeficient mice strains, such as the long-lived NOD-*scid* IL2Rgamma^null^ (NSG) mouse, which completely lacks adaptive immunity^10^, affords the opportunity to grow a wide variety of cell lines or populations in the absence of immune selection. Crucially for immunopeptidomics, the murine-derived pan-class I HLA specific antibody W6/32^11^ most commonly used for pMHC immunoaffinity purification predictably does not cross-react with murine MHC class I molecules^12^.

We selected the lymphoblastoid B-cell line JY to test our hypothesis. JY has been the subject of numerous immunopeptidome studies in the past, for a number of reasons: it is readily cultured, has high surface expression of class I HLA, and is homozygous at each of the class I loci for three alleles common in the human population (HLA-A*02:01, HLA-B*07:02, and HLA-C*07:02). The presence of HLA-B*07:02 was particularly advantageous for testing the ability of xenografts to present phosphopeptides, as the B7 allele is especially effective at presenting such sequences^13–15^.

In this study, we grew JY cells both *in vitro* and as murine xenografts and compared the immunopeptidomes and phospho-immunopeptidomes, showing that both peptides and phosphopeptides are extensively shared between both sample types, in addition to various repertoire-wide properties. This suggests that xenografts can indeed be used in place of cell culture for immunopeptidomics, extending the range and types of experiments that can be performed.

## Results

### Peptide sequences are shared across growth types

**Figure 1:**
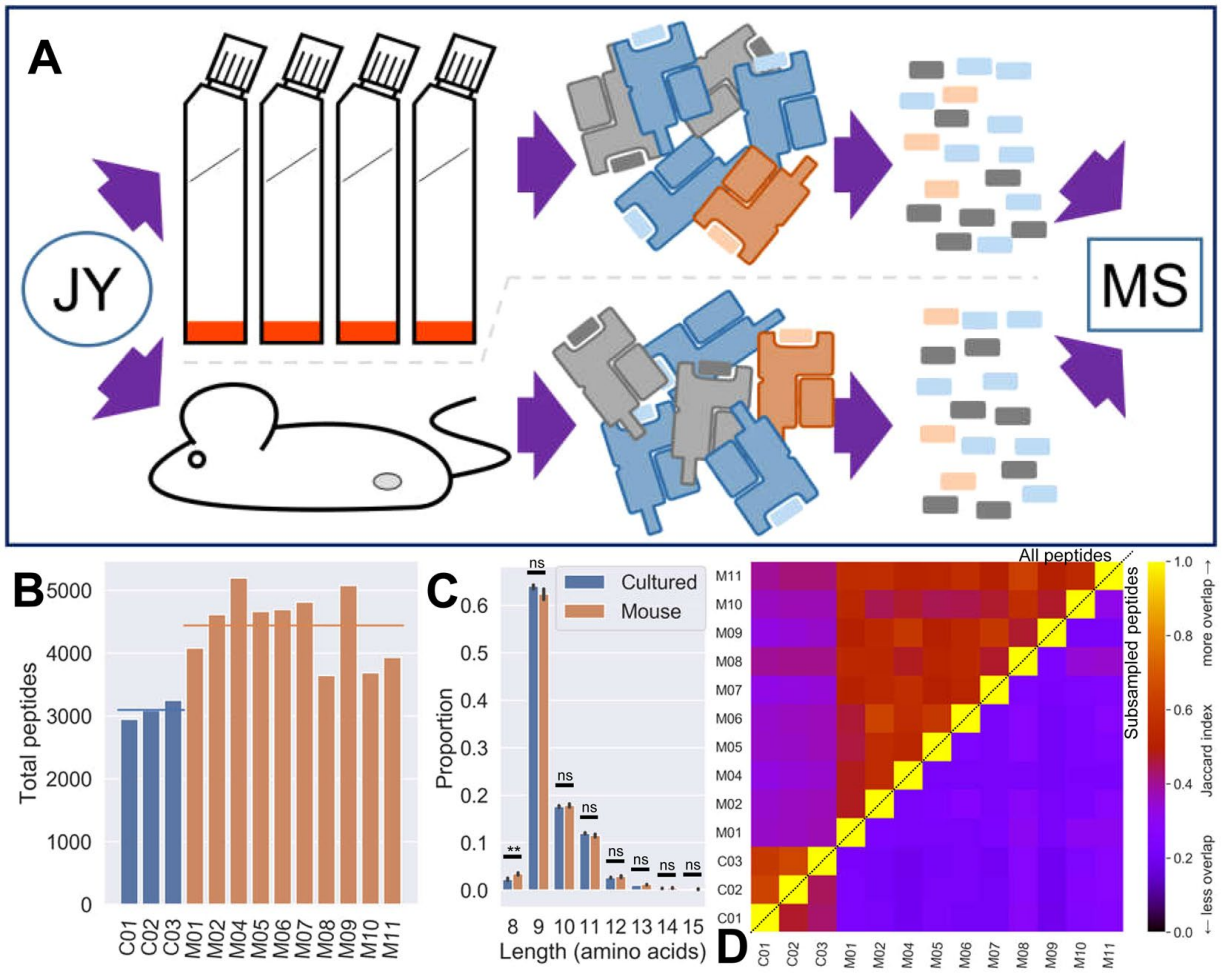
Mice as bioreactors for immunopeptidomics. (**A**) Schematic of the experiment. Cell lines (e.g. JY) were grown either via traditional *in vitro* culture, or treated as xenografts and incubated subcutaneously in immunodeficient NSG mice. Cells/tumors were then lysed, pMHC collected via immunoaffinity purification, peptides released via acid treatment and peptide sequences determined via mass spectrometry. (**B**) The total number of unique peptides obtained from each of three technical repeats of traditionally cultured JY cells (C*xx*), or one of 10 different biological repeats of murine xenograft JY cells (M*xx*). Horizontal lines indicate group means. (**C**) Length distributions of peptides produced from JY from *in vitro* or *in muridae* growth. Barplot indicates mean values plus/minus 95% confidence intervals based on bootstrapping the data. Asterisks indicate significance via Mann-Whitney U test (p > 0.05 = ns; 0.01 < p < 0.05 = *; p < 0.01 = **). (**D**) Heatmap of the Jaccard index (normalized sharing) between each pairwise comparison of samples. Comparisons of whole peptide lists shown in upper left triangle, averaged subsampled Jaccards in lower right (iterating 100 times, subsampling 2500 peptides from each file per iteration and calculating Jaccards at each iteration, plotting the mean).

In order to test our hypothesis, the well-described JY cell line was either grown in culture or as xenografts in mice, and peptides from the HLA of both sample types were detected using mass spectrometry (MS) (Fig. 1A). Broadly equivalent numbers of peptides were recovered from the three technical repeats of cultured JY cells and different biological repeats of JY xenografts (Fig. 1B, with weights of samples produced shown in Supplementary Fig. 1A). While we would expect greater variability between peptide yields from different biological versus technical samples, the yield of peptides from xenografts did not correlate with the most tumor weight (Supplementary Fig. 1B). These peptides had similar length distribution profiles, only differing in their proportions of 8-mer peptides presented (Fig. 1C).

In order to quantify the overlap of peptides between two samples we calculated the Jaccard index, a normalized measure of sharing between two sets (see Methods). The Jaccard index was calculated for each pairwise comparison of whole sample immunopeptidomes (Fig. 1D, top left triangle), revealing that while samples from each type have more overlap with repeats within their group, there are still numerous peptides shared between samples across groups. Note that a study which also compared technical replicates of JY immunopeptidomes^16^ reported overlaps which correspond to Jaccard indexes in the range 0.3 to 0.38, consistent with the values observed here even between xenograft and cultured samples. In order to overcome any biases from there being different numbers of peptides in different samples, we repeatedly subsampled the same number of peptides from each file, repeating the Jaccard calculation and averaged the results (Fig. 1D, bottom right triangle). Doing so shows us the xenograft samples are approximately as similar to the cultured samples as they are to one another.

### Xenograft JY peptidomes display similar HLA binding motifs

**Figure 2:**
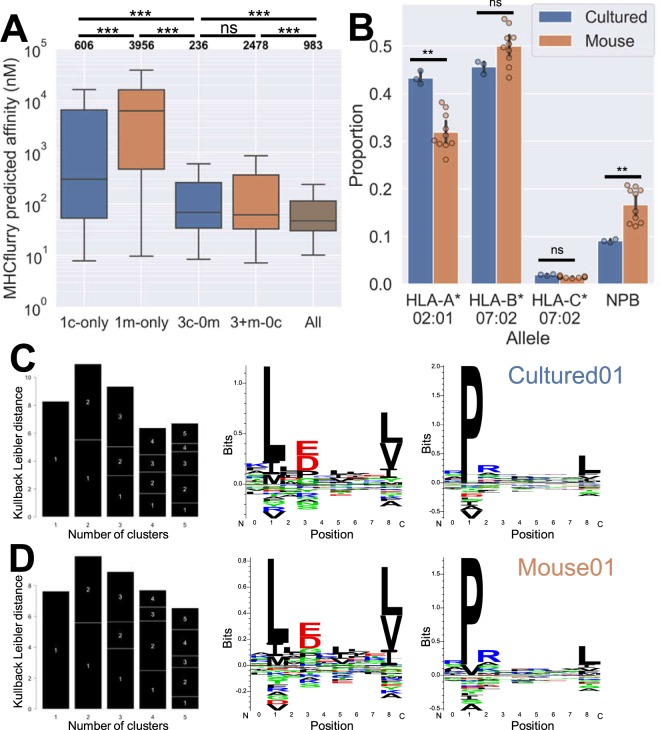
Cultured and xenograft grown JY immunopeptidomes share peptide sequences and motifs. (**A**) MHCflurry predicted nM MHC-binding affinities of peptides that appeared exclusively in: one mouse sample (1m-only); one cultured sample (1c-only); three or more mice samples and no cultured samples (3+m-0c); all three cultured but no mice samples (3c-0m); all samples. Numbers of peptides per group listed in bold above each column. Boxes indicate mean and interquartile range (IQR), while whiskers indicate the last datum under 1.5X the nearest IQR boundary. Asterisks indicate significance via Mann-Whitney U test (p > 0.05 = ns; 0.01 < p < 0.05 = *; 0.001 < p < 0.01 = **; p < 0.001 = ***). (**B**) Proportion of peptides from each sample type predicted to bind to the different HLA alleles present on JY, taking the allele with the lowest nM value as the presumed binder. Peptides with no predicted affinity < 1000 nM were deemed to have no predicted binder (NPB). Individual samples shown as dots, colored bars show the means, error bars show 95% confidence intervals based on bootstrapping the data. (**C,D**) Representative GibbsCluster-2 output of a cultured and a mouse samples’ immunopeptidome respectively. Showing Kullback Leibler distance plots (left) with two clusters maximizing the KLD, with those two clusters’ logos (right).

MHC binding characteristics were investigated by predicting peptide affinities to the HLA alleles present in JY using the MHC-binding prediction software MHCflurry^17^. Subsetting peptides based on how many of which type of sample they appeared in, we note that peptides exclusively appearing in a single mouse or cultured sample had a weaker predicted binding affinity (higher nM value) than those shared among multiple samples of the same type, with those found in a single mouse sample being significantly lower affinity than those found in a single cultured replicate (Fig. 2A). Peptides found in all samples from both growth protocols had the highest predicted affinities, indicating that sharing across groups may occur at least in part as a function of how well a peptide binds to HLA.

Peptides were assigned to the allele that most likely presented them based on which allele gave the strongest predicted affinity, with values greater than 1000 nM being classed as ‘no predicted binder’ (Fig. 2B). HLA-B*07:02 and HLA-C*07:02 – the alleles predicted to present the largest and smallest portions of peptides respectively – contributed equally across both xenograft and cultured samples. However xenograft samples showed a significant decrease in predicted HLA-A*02:01 binders, and a corresponding significant increase in peptides that were not predicted to bind to any of the relevant alleles. This was not an artefact of the software used to predict HLA binding, as the same pattern was observed when using netMHC-4.0, a different predictor^18^ (Supplementary Fig. 2A). Nor do these predicted non-binders in xenograft samples appear to be likely murine peptide contaminants; predicted non-binding peptide sequences found only in xenograft samples actually appear less often in the murine proteome than do non-binders found in both sample types or only in cultured samples (Supplementary Fig. 2B). There is also no indication that they are peptides being presented by murine class I MHC (although W6/32 should not be able to bind such molecules): predicting the affinity of these peptides as if presented by the NSG mice’s H2d MHC haplotype shows very poor predicted affinities (Supplementary Fig. 2C). The predicted non-HLA binders found exclusively in cultured samples actually showed a slightly lower predicted affinity distribution, but as with the overlap with the mouse proteome this is likely due to chance. These peptides were also derived from some of the same proteins and almost all of the same biological pathways as the non-predicted binders from cultured cells, implying that the two groups of peptides are produced in a similar manner (Supplementary Fig. 2D–F). Investigation of the electrophysical properties of these groups of non-predicted binding peptides reveals no difference in hydrophobicity, while those found exclusively in cultured cells had significantly lower charge and polarity measures (Supplementary Fig. 3).

Gibbs Cluster (version 2)^19^ was used to process each sample’s peptides, revealing extremely conserved peptide binding motifs between cultured and xenograft samples, best described with two clusters (Fig. 2C-D, and Supplementary Fig. 4). These findings mirror published JY results, being explained by the strong A2/B7 binding signatures (with the minor C7 contribution being subsumed by the similar B7 motif)^20^. This explains the strong conservation of leucine at position 2 (P2) and leucine and valine at P9 in the first column (typical of A2 nine-mers), and proline at P2 in the second column (typical of B7).

### Xenograft JY peptidomes derive from similar proteins and pathways

**Figure 3:**
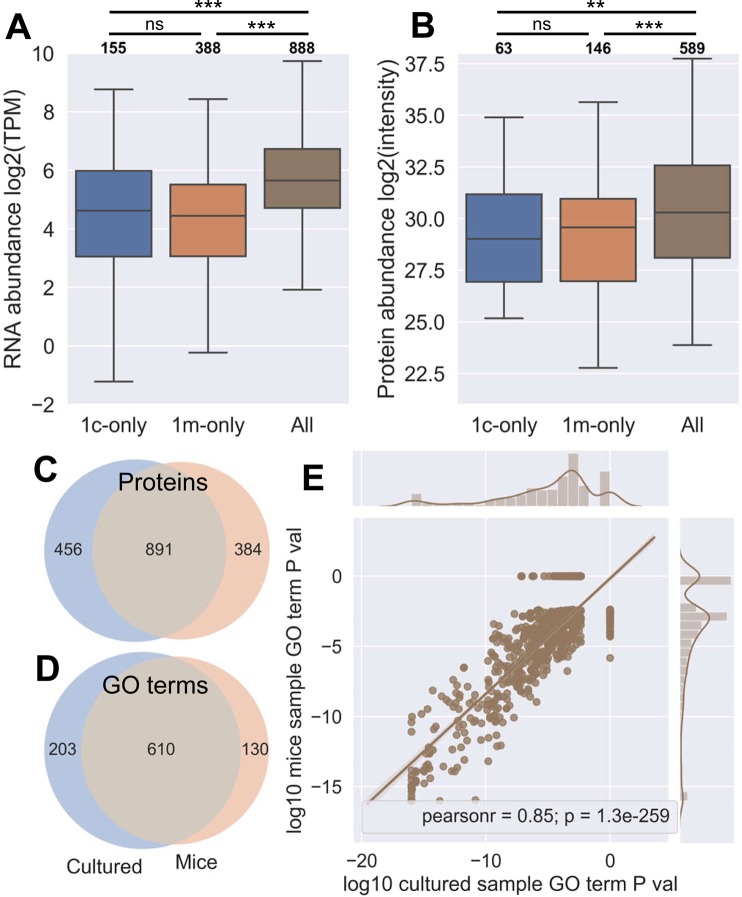
Cultured and xenograft grown JY immunopeptidomes derive from similar proteins and pathways. (**A**) Abundance of transcripts whose proteins had detectable peptides in: exclusively one cultured sample (1c-only), one mouse (1m-only), or in all samples, as present in publicly available JY RNAseq data. Log2 transcripts per million (TPM) from Kallisto analysis of raw FASTQ sequence data shown on the y axis. Numbers of transcripts per group listed in bold above each column. (**B**) As in **A**, but for publicly available proteomic data, with log2 mass spectrometric intensity on the y axis. Numbers of proteins per group listed in bold above each column. Asterisks in **A** and **B** indicate significance via Mann-Whitney U test (p > 0.05 = ns; 0.01 < p < 0.05 = *; 0.001 < p < 0.01 = **; p < 0.001 = ***). (**C**) Overlap of proteins represented by peptides in the immunopeptidomes of all cultured repeats (left, blue) or all mouse samples (right, orange). (**D**) As in **C**, but showing overlap in enriched GO terms of proteins featured, determined by overrepresentation analysis via WebGestalt (FDR < 0.05). (**E**) Linear regression of the P values of GO term enrichment of terms shown in **D**. GO terms that were not shared were assigned a P value of 1, while those with a value of 0 were rounded down to the nearest order of magnitude (1 × 10^–16^) for plotting on logged axes.

In addition to testing whether the peptide properties are conserved between xenograft and *in vitro* growth, we assessed whether the different growth types might select for peptides derived from different proteins or pathways. Mapping peptides to their source proteins and comparing with published data revealed that peptides found in all samples tended to be derived from more abundant transcripts (Fig. 3A) and proteins (Fig. 3B) than those that were found only in a single mouse or cultured replicate. By binarizing the presence/absence of a protein we plotted correlations with the published transcriptomic and proteomic abundances. Almost all samples were strongly correlated for both, with stronger correlations for those grown *in vitro* (Supplementary Figs 2 and 6). Note that the published RNAseq and proteomic data come from JY grown traditionally in culture.

Comparison of the proteins that were represented in the immunopeptidomes of both all cultured and all xenograft samples revealed a substantial overlap (Fig. 3C). Calculation of over-represented GO terms in each group’s proteins relative to the whole proteome revealed an even greater overlap (Fig. 3D), with the P values of the enriched GO terms in each analysis being highly correlated (Fig. 3E). The distribution of various biological parameters in the over-representation analysis were also highly conserved between both cultured and mouse grown JY samples (Supplementary Fig. 7).

Therefore not only are xenograft grown JY recapitulating the broad immunopeptidome characteristics (and most of the same actual peptide sequences), they are doing so by drawing on the same proteins and pathways.

### Xenograft-derived peptides are consistent with published JY immunopeptidomes

**Figure 4:**
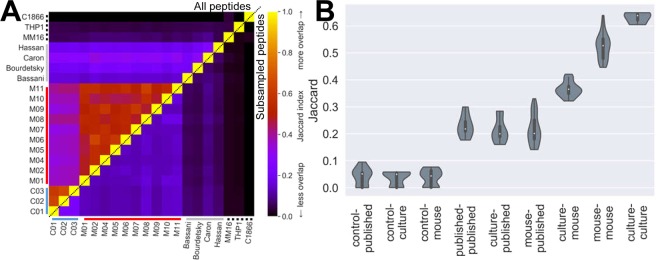
Xenograft JY immunopeptidomes are even similar to published cultured datasets. (**A**) Heatmap of Jaccard indices showing overlap of immunopeptidomes between cultured JY (see blue axes bars), mouse grown JY (red), published cultured JY (grey) and published non-JY cells (dotted black). Top-left triangle indicates comparisons of all peptides per sample, bottom-right triangle indicates the average Jaccard from subsampling 1500 peptides per sample 100 times. Control peptidomes are: a melanoma tumor (MM16), a monocyte-like acute monocytic leukemia line (THP1), and a T lymphoblastoid line (C1866). (**B**) Violinplot of grouped pairwise comparisons shown in **A**, ignoring redundant comparisons or samples compared against themselves. White central dots show the median, thick gray bars the interquartile range, thin gray lines the 95% confidence interval, and violin spread shows a kernel density estimation of the data distribution.

The experiments presented so far are somewhat confounded in that we are comparing xenograft biological replicates against cultured technical replicates (imposed by the burden of culturing sufficient cells for assessment). To circumvent this problem, we performed pairwise analyses of our JY *in vitro* and *in muridae* grown cells against published JY immunopeptidomes from other laboratories (all produced through standard cell culture), as well as to other non-JY cell lines and tissues taken from the SysteMHC resource^21^ (Fig. 4).

Taking complete peptide lists, we observed that JY immunopeptidomes produced from either xenografts or from culture had equal overlap with previously published JY immunopeptidomes as with one another (Fig. 4A, top left triangle, and 4B). In fact, they were as similar to published JY immunopeptidomes as those four samples – each from different groups using their own methodologies – were similar to each other. When looking at averaged overlap between size-matched subsamples of equal numbers of peptides, our cultured JY had slightly more overlap with published data (Fig. 4A, bottom right triangle, and Supplementary Fig. 8). All JY samples shared significantly fewer peptides (less overlap/lower Jaccard index) with control immunopeptidome samples that would be expected to have minimal overlap (on the basis of sharing few to no HLA alleles and being derived from different cell types). Thus, our xenograft grown JY immunopeptidomes are as good at recapitulating a published JY immunopeptidome as any other group’s cultured cells.

### JY Xenograft phospho-immunopeptidomes are consistent with cultured JY

Having recapitulated the broad immunopeptidome we wanted to assess whether xenografts could reproduce the phospho-immunopeptidome, the fraction of the immunopeptidome which retains phosphorylated residues. This subset contains a number of shared cancer neoantigens which could be of great immunotherapeutic use^14,22^, yet is technically harder to study due to its lower surface abundance. Phosphopeptides were extracted from a subset of eluted JY immunopeptidomes via immobilized metal affinity chromatography (IMAC) and determined by MS. The addition of phosphostandards in this section of the protocol allowed us to estimate relative abundances (see methods), adding an extra layer of information compared to the non-phospho-immunopeptidomes.

**Figure 5:**
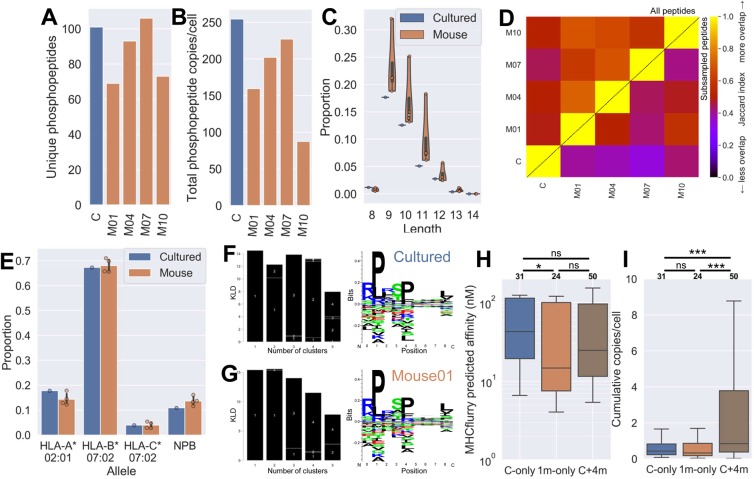
Phospho-immunopeptidomes are similar between *in vitro* and xenograft grown cells. (**A**) Total number of different phosphopeptides per sample. (**B**) Total inferred cumulative abundance (copies per cell) of phosphopeptides per sample. (**C**) Length distribution of phosphopeptides detected in the four mouse grown samples, and the single cultured sample. (**D**) Jaccard index heatmap of phosphopeptide sequences found across the mouse and cultured samples. Whole phosphopeptide list comparisons shown in top left triangle, subsamples of 50 phosphopeptides averaged from 100 iterations shown in bottom right. (**E**) Proportion of peptides from each sample type predicted to bind to the different HLA alleles present on JY (using the non-phosphorylated equivalent sequence), taking the lowest nM value as the binding allele. Peptides with no predicted affinity < 1000 nM were deemed to have no predicted binder (NPB). (**F-G**) Representative GibbsCluster-2 output of the cultured and a mouse sample phospho-immunopeptidome respectively. Showing Kullback Leibler distance plots (left) with one cluster maximizing the KLD (logo on right). (**H**) MHCflurry predicted MHC-binding affinities of peptides that appeared exclusively in: the one cultured sample (C-only); one mouse sample (1m-only); or in all samples (C+4 m). Numbers of peptides per group listed in bold above each column. (**I**) Cumulative abundance (copies/cell) of peptides from groups described in **H**.

Approximately similar numbers of phosphopeptides were detected on cultured and xenograft derived JY cells (Fig. 5A). Cumulatively the cultured sample’s phosphopeptides were more abundant than those of the mice (Fig. 5B), although only marginally compared to some samples. The length profiles were again broadly equivalent, although the cultured sample contained more 8-mers and fewer 9-11-mers proportionally (Fig. 5C). We again saw considerable overlap of phosphopeptide sequences between the cultured and xenograft samples, although the mice samples are more similar to one another than to the cultured sample (Fig. 5D and Supplementary Fig. 9). Moreover, the abundance of the phosphopeptides between any two samples correlated highly (Supplementary Fig. 10). Note that the low R values may in part be explained by the copies/cell value calculation being confounded in the mice samples due to the presence of mouse-derived tissues contributing to tumor weight but not to JY peptidomes. UpSet analysis was used to visualize the intersection of phosphopeptides across the multiple samples^23^. This revealed that the largest single set of unique phosphopeptides contains those shared among all samples, with the two next largest sets being those specific to the two samples with the largest number of unique samples (the cultured sample and M07, Supplementary Fig. 11).

MHC prediction algorithms are currently unable to account for differences in binding affinity which can occur as a consequence of post-translational modifications such as phosphorylation. Therefore in this study we predicted affinity for the corresponding unmodified sequence, acknowledging that this cannot predict the impact on affinity that such phosphorylation events can incur^22,24^. The predicted HLA allele binding contributions and MHC-bound phosphopeptide motifs were almost indistinguishable between *in vitro* and *in muridae* grown samples (Fig. 5E–G, and Supplementary Fig. 12). There did not seem to be an enrichment of high affinity binding among the highly shared phosphopeptide sequences (Fig. 5H) as there was for whole immunopeptidomes (Fig. 2A). However phosphopeptides restricted to a single mouse sample did similarly show a higher-affinity skew relative to those in the single cultured sample. Importantly, we observed that phosphopeptides shared among all samples had a significantly higher abundance on the cell surface (Fig. 5I).

### Xenograft samples match cultured immunopeptidomes in other cell lines

In order to evaluate the broader applicability of the technique we extracted immunopeptidomes from two additional cancer cell lines: MDA-MB-436 (breast) and Colo205 (colorectal). Cells were grown fresh for both culture and xenograft conditions for MDA-MB-436, whereas xenograft-grown Colo205 peptides were compared against the peptides of cultured cells that had been grown years previously, before pMHC extraction and cryopreservation. This produced samples with equivalent numbers of peptides, again showing the typical pattern of MHC-bound peptide length distributions (Supplementary Fig. 13A-B).

**Figure 6:**
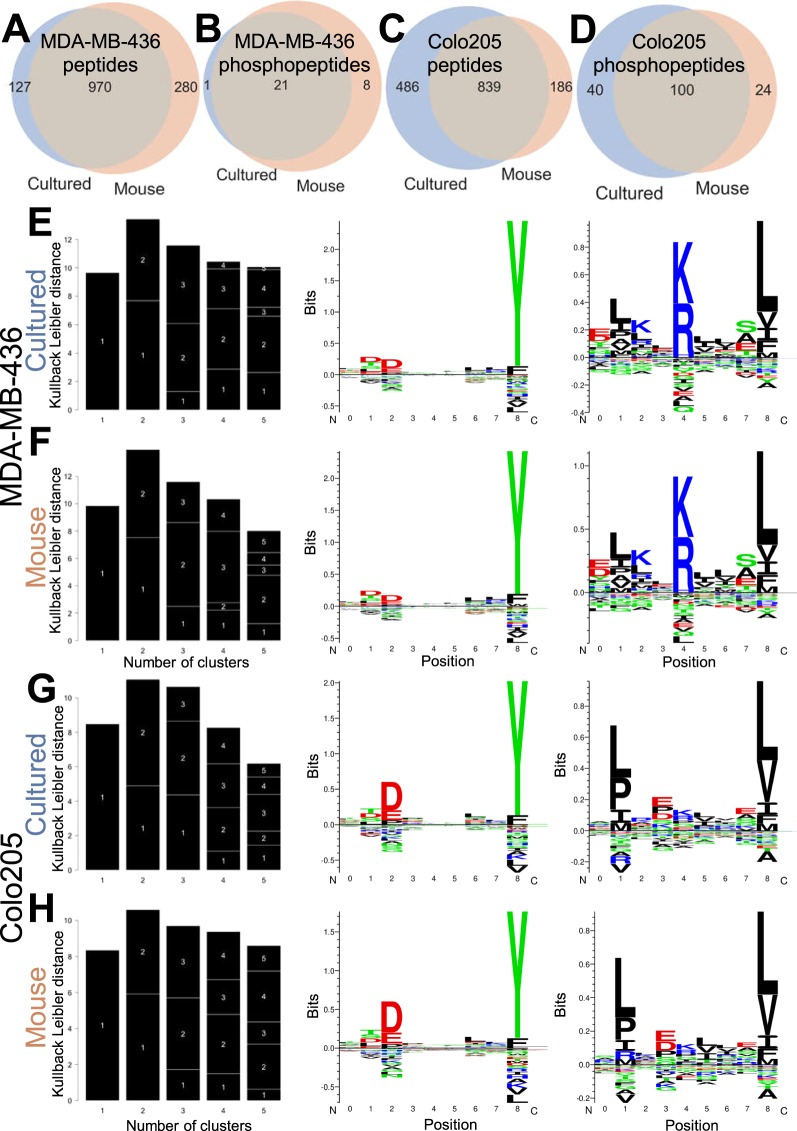
Immunopeptidomes are similar between *in vitro* and xenograft grown cells of other cell lines. (**A**-**B**) Overlap of standard and phospho-enriched immunopeptidomes respectively for MDA-MB-436 breast cancer cells grown in culture (blue) or as a xenograft (orange). (**C-D**) Similar overlap of the cultured/xenograft immunopeptidomes and phospho-immunopeptidomes respectively for the colorectal cancer cell line Colo205. (**E**-**F**) GibbsCluster-2 output of the Kullback-Leibler distance (left) and two-cluster logos for MDA-MB-436 cells grown in culture (**E**) or in a mouse (**F**). (**G**-**H**) As in **E**/**F**, but for Colo205 cells.

The standard and phospho-enriched immunopeptidomes of both cell lines showed a strong degree of overlap, with the majority of all peptides and phosphopeptides in either growth mode being found in the other (Fig. 6A–D). (Note that the paucity of MDA-MB-436 phosphopeptides relative to the other lines is likely best explained by the lack of an allele which readily presents them, such as the HLA-B*07:02 allele JY and Colo205 express). Furthermore the motifs of the peptides found on each cell line was extremely consistent between growth types (Fig. 6E–H), with both cell lines showing a strong HLA-A*01 motif along with either an HLA-B*08 or HLA-A*02 motif. These observations are further supported when using MHCflurry to assign putative responsible binding alleles, as the majority of peptides are predicted to be presented by those alleles in their respective cell lines, to broadly similar degrees between cultured and xenograft grown cells (Supplementary Fig. 13C). We also note that in these cell lines there is no obvious difference in the proportion of peptides that were not predicted to bind any of the relevant HLA alleles as observed with JY.

Collectively these data show that xenograft grown cells produce highly similar immunopeptidomes and phospho-immunopeptidomes to those grown in culture, likely preferentially representing those antigens that are ordinarily presented at higher frequency or at higher affinity.

## Discussion

The field of immunopeptidomics offers researchers unparalleled insights into the displayed peptide antigens which ultimately underlie adaptive immune responses. This not only aids our understanding of basic immunology and cell biology, but presents opportunities to select neoantigens for vaccination and other immunotherapeutic interventions^4,25,26^. Despite innovative efforts to streamline sample processing to maximize yield^1^, immunopeptidomics is still limited by requiring sufficient material for detection by mass spectrometry. As with all complex biological mixtures of unknown content, the only way to get more material to study is to acquire more of the cells or tissue of interest. This imposes a technical bias on the number and nature of samples which can be easily processed, e.g. favoring cell lines that can be grown rapidly in culture, or tumors which present with large resectable masses. This property is even more true when aiming to study specific portions of the immunopeptidome, such as those bearing post-translational modifications; as they account for only a fraction of the total immunopeptidome they require a correspondingly larger mass of cells to detect equally well.

Murine xenografts have been adopted across many fields as a means to study numerous aspects of human cellular biology *in vivo*. They allow rapid growth of a variety of cell lines, even from low initial input cell numbers such as from circulating tumor cells^27^. Moreover, with respect to studying cancer, xenografts can be argued to better recapitulate the complex biology of tumor cell growth than simple 2D or even 3D culture^28^. We therefore hypothesized that this process could be used to help surmount that challenge of immunopeptidomics, the struggle to attain enough material, while maintaining utility and biological relevance. To test our hypothesis, we grew the JY cell line either via traditional *in vitro* cell culture, or through xenograft growth in immunodeficient NSG mice. Note that while we are aware of at least one other group employing immunodeficient mice to grow cancer cell lines for immunopeptidomics^29^, we believe that we are the first to perform a head-to-head comparison with traditional *in vitro* growth.

We found that xenograft grown JY cells recapitulated the peptide repertoires of cultured cells remarkably well, producing peptide lists with considerable overlap, and sharing several important immunopeptidome properties. Peptides shared between both growth methods seemed to be enriched for high-affinity binders to MHC, or were derived from relatively more abundant transcripts and proteins. Our cultured and xenograft grown JY immunopeptidomes were also as similar to other groups’ previously published examples as those published immunopeptidomes are similar to each other. This alone suggests that the method is at least as effective as replicating JY immunopeptidomes as conventional culture. Repeating the protocol with two cell lines of entirely different tissue origins produced immunopeptidomes with comparable overlap between culture and mice, suggesting that the protocol could be robust across a variety of cancer types.

There were a small number of properties which were not identical between *in vitro* and ‘*in muridae*’ grown JY cells. Perhaps the most notable difference was the greater population of peptides found on xenograft-grown JY cells that were not predicted to bind any of the HLA alleles present on the cell line. Despite control analyses failing to attribute them to a common or easily explained source of noise, these discrepancies could be perceived as a failure of mice implantation to fully reconstitute the exact composition of cultured JY. Perhaps some artefact of the xenograft process itself skews the immunopeptidome in this line. However, a possibility exists that artefacts or biases have actually been introduced to the field in part by relying on peptides eluted from *in vitro* cultured samples, which would then filter down to current models and *in silico* prediction algorithms. For example, perhaps the differences in cellular interaction, extracellular milieu composition, and homeostatic versus periodic nutrition (i.e. changing media) predispose certain peptides to be lost in culture, which can be rescued by using murine bioreactors. While an interesting possibility – perhaps somewhat more plausible given the different charge and polarity distributions observed between these groups of peptides – this study was not designed to assess whether this was the case. On a pragmatic level the differences between the peptide distributions between the growth types may be so minor as to not matter for many applications, and indeed this phenomenon was not observed in the other two cell lines tested.

We also noticed some variability in number of peptides recovered from each xenograft, which did not seem to correlate with the input weight of xenograft tissue; if anything, the largest samples gave the fewest peptides. While it was not controlled for in this study, it seems likely that a differential contribution of murine tissues (e.g. vasculature, stroma, etc.) to the mass of each tumor could be the key determinant of this disconnect. Use of xenografts might therefore benefit from a more rigorous normalization strategy, perhaps using some proteomic or PCR based assay to quantify mouse versus human contribution to the samples.

Study of specific post-translationally modified peptides perhaps stands the most to gain from techniques designed to increase material yield, as these peptides occupy such a small fraction of all the molecules presented. We looked at the conservation of phosphopeptides between cultured and xenograft grown cells, again revealing considerable sequence- and whole-immunopeptidome-level overlap.

Interestingly, despite the lack of evidence in our data for murine peptides featuring in the bulk immunopeptidomes of our xenograft-grown cells, there was a notable singular exception detected in the enriched phosphopeptides. RQPsIELPSM (with the lowercase ‘s’ denoting a phosphoserine) was found in all of the xenograft samples at some frequency – or at the very least an ion with an identical mass and retention time – and not in their cultured counterparts. While this sequence is not known to be in the human proteome it is found in the mouse, in Lymphocyte-Specific Protein 1 (LSP1). Furthermore according to the PhosphoSitePlus resource^30^ this serine is known to be phosphorylated in mice. However its source in this dataset still presents some mysteries. Hypotheses as to its cellular source are straightforward: while these NSG mice do lack a number of immune cells, LSP1^+^ monocytes and neutrophils remain, as well as the potential for LSP1^+^ endothelial cells^31^, providing potential xenograft-proximal sources. Its molecular source is less clear. It could theoretically come from dissociation off murine MHC it had been presented on, although it is not predicted to bind to any of the mouse H-2 MHC alleles (using either of the predictors employed here, NetMHC 4 and MHCflurry). Intriguingly this phosphopeptide was detectable in the spectra of the majority of the tumor and normal tissue samples from the recent draft murine immunopeptidome project^32^. This does not address the source of this peptide in our data however. In that study the peptide was only detectable when using the anti-H-2D^b^ antibody B22–249.R1 for the MHC pulldown (and not the anti-H-2K^b^ antibody Y-3); however the NSG mice used in our study express H-2D^d^ which is not bound by this antibody^33^. The non-phosphorylated equivalent peptide is weakly predicted to bind to HLA-B*07:02, but this also does not explain its presence in so many of the xenograft samples as not all of the tested lines (e.g. MDA-MB-436) express this allele. Perhaps this phosphopeptide is made by some murine cells in such quantities that it is able to make it to the extracellular space and passively load onto the graft’s HLA molecules – potentially aided by a phosphorylation-induced boost in affinity not covered by modern predictors. Regardless of the origin of this contaminant, it seems to be the sole example of a peptide directly attributable to a mouse sequence; given the number of peptides found we still believe this to be a robust and useful methodology.

This study focused on JY, a strong contender for the cell line with the most-studied immunopeptidome to date, as an example to test our hypothesis. It has common and homozygous HLA alleles, which are expressed to a high level, and grows readily in culture. In cases such as this, there is certainly less need for a solution like murine xenografts, as cells can be grown in culture in comparable time frames. Many lines grow far slower than JY in culture however, and have lower expression of HLA alleles, potentially with weaker binding affinity distributions. In such cases production of sufficient material by culture can become extremely impractical if not impossible, as operator time and tissue culture equipment availability can become limiting. These lines are probably those that would benefit most from xenograft sample production, particularly in experiments where a large panel of lines are to be grown simultaneously.

It should be noted that use of mice for immunopeptidomics requires additional specialized facilities, trained staff, and regulatory and ethical procedures, in addition to the typical cell culture capacity requirements. However given the widespread adoption of murine studies across the fields of immunology and cancer research we expect that many groups should have access to such facilities, either locally or through collaboration. This methodology may also be incorporated into existing studies, e.g. those which explore PDX tumor growth but which don’t currently use up all of the tissue. Given the repeatability of this method on the Colo205 cell line – whose cultured sample in this experiment was processed and had peptides frozen approximately eight years prior to the generation of the xenograft sample, yet still showed remarkable immunopeptidome conservation – it is possible that a much greater range of experiments than our groups personally have imagined will be possible. Indeed, we anticipate that this methodology will help the field of immunopeptidomics as it seeks to expand into higher-throughput and more diverse applications.

## Methods

### Cell culture

JY cells were grown in RPMI 1640 supplemented with 10% fetal bovine serum (FBS, Gibco #26140–079) or newborn bovine serum (NBS, Gemini Bio-Products #100–504), 2 mM/1% GlutaMAX (ThermoFisher Scientific #35050061), and 1% penicillin-streptomycin. MDA-MB-436 were cultured in DMEM/F12, similarly supplemented, passaged via trypsinization (retaining cells still in suspension). Cells were incubated under standard conditions (37 °C, humified, 5% CO_2_), and split three times a week keeping cell numbers in the range 0.5–1 × 10^6^/ml.

When culturing cells for direct pMHC isolation, cells were obtained by harvesting cells during serial subculture. Cells were washed twice in PBS, counted, and pelleted in pre-weighed conical centrifuge tubes. Pellets were snap frozen in liquid nitrogen, tubes reweighed to obtain pellet weight, before storage at −80 °C.

The TRON Cell Line Portal^34^ was used to obtain HLA allele information for the cell lines used in this study.

### Animals

All animal procedures were performed in accordance with Federal and Institutional Animal Care and Use Committee requirements, under a protocol approved at Massachusetts General Hospital. NOD-*scid* IL2Rgamma^null^ (NSG) mice (Jackson Laboratories) were subcutaneously engrafted with cells in Matrigel (Corning) according to the manufacturer’s instructions. 5 × 10^6^ cells were injected per mouse. Tumor burden was monitored daily. Once tumor size reached 200 mm^3^, mice were euthanized with CO_2_, and tumors were quickly collected and snap frozen in liquid nitrogen before storage at −80 °C.

### pMHC isolation

MHC-bound peptides were isolated as described previously^6,14^, described here in brief. First, the pan-human class I antibody W6/32 (#93134, BioLegend) is conjugated to NHS-sepharose beads (GE, #17–0906–01). 3 mg of antibody is prepared per 1 × 10^9^ cells or 1 g of tissue, whichever is greater. 100 μl of beads are used per 1 mg of input antibody. Beads are washed twice in PBS and incubated rotating overnight with antibody. The following day beads are pelleted, blocked for 1 hour with 0.1 M Tris-HCl, then washed twice in alternating solutions of 0.1 M acetate/0.5 M NaCl and 0.1 M Tris-HCl. Beads are resuspended in 20 mM Tris/150 mM NaCl up to 1 mg/ml remaining antibody and stored at 4 °C until use.

Cells are lysed the following day in lysis buffer (either with 5 mM EDTA, pH 8 for JY tumors, or with 20 mM Tris-HCl, 150 mM NaCl, 1% CHAPS, pH 9 for all other samples), using 5 ml per 1 × 10^9^ cells or 1 g of tissue. Lysis buffer was supplemented with protease and phosphatase inhibitor cocktails, all sourced from Sigma Aldrich: the JY tumor samples were lysed with phosphatase inhibitor cocktail II and III (#P5726 and #P0044), aprotinin (#A4529), leupeptin (#L2884) and pepstatin A (#77170); all other samples were lysed with PhosSTOP phosphatase inhibitor and cOmplete mini protease inhibitor (#4906845001 and #11836153001 respectively). Cells are lysed from frozen, rotating in buffer at 4 °C for 1–2 h. Prior to lysis, JY tumor samples were dissociated using a gentleMACS (Miltenyi Biotech), while the MDA-MB-436 breast cancer tumor sample was powdered using a nitrogen-cooled tissue pulverizer (Cellcrusher). Lysed cells are ultracentrifuged at 36,800 rpm for 1 hour at 4 °C, and supernatants are then mixed with the antibody-bead conjugates prepared earlier and rotated at 4 °C overnight to allow MHC binding.

The following day, pelleted beads are washed in 10 ml lysis buffer, resuspended in ~500 μl 20 mM Tris-HCL and transferred to a microcentrifuge tube or Poly-prop column. Being kept at 4 °C, beads are then washed with: 2 × 20 mM Tris-HCL, 150 mM NaCl; 2 × 20 mM Tris-HCl, 1 M NaCl; 3 × 1 ml 20 mM Tris-HCl. Washed beads are then spun through a pre-wet Amicon Ultra centrifugal filter column ( >  = 3 kDa, Millipore), and all liquid is removed by centrifugation. At this point columns are parafilmed and stored at −80 °C for later processing.

### Sample Desalting via STop And Go Extration (STAGE) Tips

Dry bead-bound HLA:peptide complexes contained within the Amicon filters were sent for STAGE tip cleanup methodology. STAGE tips were fabricated as previously described^35^ and each stage tip was used for desalting of 4E8 to 6E8 C.eq. of sample. Fabricated STAGE tips were equilibrated using the following wash steps: two 1 minute washes of 100 μl of methanol at 3500 × g, one 1 minute wash of 50 μl of 80% acetonitrile/0.01% acetic acid at 1500 × g, and two 1-minute washes of 100 μl of 1% acetic acid at 3500 × g. Thawed beads were transferred from the filter to a separate low-protein binding tube using subsequent water rinses to ensure complete transfer. Beads were centrifuged at 300 × g for 1 minute and the supernatant was loaded onto the STAGE tip in 150 μl aliquots for 1 minute at 3500 × g. Beads were washed using 100 μl of 3% acetonitrile/5% acetic acid followed by 50 μl of 1% acetic acid and supernatants were loaded onto STAGE tips for 1 minute each at 3500 × g.

For the elution of peptides from HLA molecules bound to beads, 150 μl of 10% acetic acid was added to the tube which was then shaken for 5 minutes at room temperature. The beads were centrifuged at 300 × g for 1 minute and the supernatant transferred to a low-binding tube. This process was repeated to ensure complete elution of peptides from HLA molecules and the supernatant added to the low-binding tube. Two internal phosphopeptide standards were spiked into the 10% acetic acid elution supernatant at 250 fmol each. Then, the elution supernatant was sonicated for up to 3 seconds prior to loading onto the STAGE tips. The elution supernatant was loaded onto the STAGE tips in 150 μl aliquots at 3500 × g until the entire volume had passed through. STAGE tips were washed using three rounds of 100 μl of 1% acetic acid. Peptides were eluted from the STAGE tips using the following stepwise gradient of increasing acetonitrile concentrations: 20 μl of 20% acetonitrile/0.1% acetic acid, 20 μl of 40% acetonitrile/0.1% acetic acid, and 20 μl of 60% acetonitrile/0.1% acetic acid. Eluted peptide fractions were dried to completion using a Centrivap, reconstituted in 0.1% formic acid to a concentration of 1E7 cell equivalents per μl and stored at −80 °C until further use.

### Non-enriched immunopeptide MS

5E7 C. Eq. of each sample was loaded onto a fused silica microcapillary analytical column (360 μm o.d. × 75 μm i.d.) equipped with a picofrit, a 10 μm electrospray tip, and packed with 20 cm of 1.9 μm C18 Reprosil resin and connected to an HPLC. Peptides were eluted using an HPLC gradient of 0–30% Solvent B (80% acetonitrile in 0.1% formic acid) in 90 minutes at a flow rate of 200 nl/min and electrospray ionized directly into an Orbitrap Fusion Lumos Tribrid mass spectrometer. Full-scan high resolution mass spectra (MS1) were acquired in the Orbitrap mass analyzer and MS/MS spectra (MS2) were acquired using HCD and ETD fragmentation methods in the linear ion trap of the instrument. A top-speed method was used in which one high resolution MS1 scan (resolving power of 60,000 at 400 m/z) was first acquired, followed by selection of as many parent ions as possible in a two second timeframe (in order of decreasing abundance) for fragmentation by HCD and ETD. Data-dependent parameters included a repeat count of 3, repeat duration of 10 seconds, and exclusion list duration of 10. Additionally, ions with a charge state of +1 were excluded. Additional parameters included a pre-calibrated ETD reaction time, FTMS automatic gain control (AGC) target of 2e5 charges, ITMS AGC target of 1e4 charges, and an ETD reagent target of 1e4 charges.

The analysis of the non-enriched immunopeptide from MDA-MB-436 samples were different in some parts. 2 E7 C.eq of samples were loaded onto a fused silica microcapillary pre-column (360-µm o.d. × 75-µm i.d.) equipped with a 2-mm Kasil 1624 frit and packed with 10 cm of C18 reversed-phase packing material (5–20 µm diameter, 120 Å pore size).The analytical column (360 μm o.d. × 50 μm i.d.) was packed with regular C18 (5 μm diameter) packing material and equipped with a laser-pulled, electrospray emitter tip (2 μm diameter). Peptides were eluted with Solvent B consisted of 66% acetonitrile in 0.1% acetic acid, eluted at 60–100 nl/min, processed on a Thermo Scientific™ Orbitrap Fusion Tribrid Mass Spectrometer. The same top-speed method and data-dependent parameters was used for MS1 scan followed by the selection of as many parent ions as possible for fragmentation by CAD for +2 and fragmentation by ETD for +2 and +3 charge states. High-resolution ETD and CAD for +3 and +2 ions respectively were accumulated up to an AGC target value of 2E5 with a maximum injection time of 120 ms and were detected in the Orbitrap analyzer at a resolution of 15k. Additional parameters for complimentary low-resolution ETD for +2 ions were the same as JY.

### Phosphopeptide enrichment and MS

Samples were subjected to a Fischer esterification and phosphopeptide enrichment via immobilized metal affinity chromatography as previously described^36^.

Briefly, a fused silica microcapillary IMAC column (360 μm o.d. × 75 μm i.d.) equipped with a 2 mm Kasil 1624 frit was packed with 5.5 cm of POROS MC 20 iminodiacetate resin. The IMAC column was pressure rinsed at a flow rate of 20 μl/min with the following steps: a 20 minute water rinse, a 10 minute 50 mM EDTA rinse, and a 10 minute water rinse. Three rounds of activation were performed using 100 mM FeCl3 for 5 minutes at a flow rate of 20 μl/min followed by a 3 minute incubation The activated column was equilibrated with 25 μl of 0.01% acetic acid at a flow rate of 0.5 μl/min. The dried, esterified peptide sample was reconstituted in 50 μl of 1:1:1 (methanol:acetonitrile:0.01% acetic acid, vol/vol) and pressure loaded onto the activated IMAC column at a flow rate of 0.5 μl/min. Following sample loading, 25 μl of 1:1:1 was added to the sample tube and loaded onto the IMAC column at a flow rate of 0.5 μl/min. A final rinse with 15 μl of 0.01% acetic acid at a flow rate of 0.5 μl/min was completed.

A fused silica microcapillary pre-column (360 μm o.d. × 75 μm i.d.) equipped with a 2 mm Kasil 1624 frit and packed with 8 cm of 3 μm C18 Reprosil resin was butt-connected to the IMAC column via a Teflon sleeve and enriched phosphopeptides were eluted onto the pre-column by pressure loading freshly-prepared 250 mM L-ascorbic acid in water (pH 2) at a flow rate of 0.5 μl/min for 60–90 minutes. A final rinse of 0.01% acetic acid at the same flow rate was completed prior to disconnection of the pre-column from the IMAC column. The pre-column was connected to a fused silica microcapillary analytical column (360 μm o.d. × 75 μm i.d.) equipped with a picofrit, a 10 μm electrospray tip, and packed with 20 cm of 1.9 μm C18 Reprosil resin and connected to an HPLC.

JY phosphopeptides were eluted using an HPLC gradient of 0–60% Solvent B (80% acetonitrile in 0.1% formic acid) over 60 minutes at a flow rate of 200 nl/min and electrospray ionized directly into an Orbitrap Fusion Lumos Tribrid mass spectrometer. Full-scan high resolution mass spectra (MS1) were acquired in the Orbitrap mass analyzer and MS/MS spectra (MS2) were acquired using CAD and ETD fragmentation methods in the linear ion trap of the instrument. A top-speed, neutral-loss triggered method was used in which one high resolution MS1 scan (resolving power of 60,000 at 400 m/z) was first acquired, followed by selection of as many parent ions as possible in a three second timeframe (in order of decreasing abundance) for fragmentation by CAD. If a neutral loss characteristic of the loss of phosphoric acid (Δ98 Da) was present in the CAD spectrum among the top eight product ions, an ETD scan of that precursor was then acquired. Data-dependent parameters included a repeat count of 3, repeat duration of 10 seconds, and exclusion list duration of 10. Additionally, ions with a charge state of +1 were excluded. Additional parameters included a pre-calibrated ETD reaction time, FTMS automatic gain control (AGC) target of 2e5 charges, ITMS AGC target of 1e4 charges, and an ETD reagent target of 1e4 charges. MDA-MB-436 phosphopeptides were processed similarly, except Solvent B consisted of 66% acetonitrile in 0.1% acetic acid, eluted at 60–100 nl/min, processed on an Orbitrap Fusion.

### MS data analysis

Data analysis was performed using Xcalibur software (Thermo Electron Corporation). For standard immunopeptides, raw data files were searched using Byonic (Protein Metrics)^37^ against the Swissprot human protein database with the following parameters: no enzyme specificity, variable modifications of oxidation (methionine, tryptophan, cysteine), cysteinylation (cysteine), and phosphorylation (serine, threonine, tyrosine), ± 10 ppm precursor mass tolerance ( ± 6 ppm for MDA-MB-436), ± 0.40 Da product ion mass tolerance, and 1% FDR. This additionally outputs the source protein of each peptide. For the non-enriched immunopeptide from MDA-MB-436 samples ± 6 ppm precursor mass tolerance and ± 20 ppm product ion mass tolerances were considered in the Byonic search. Phosphopeptide searches also used the fixed modifications of methyl esters (aspartic acid, glutamic acid, C-termini) parameter. Hits from both searches were used to guide the analysis and phosphopeptide sequences were determined by accurate mass measurement and manual interpretation of MS2 spectra for *de novo* sequencing. Raw data files were also searched against an in-house phosphopeptide database containing all identified phosphopeptides from previous samples using the same parameters. All phosphopeptide sequences were manually validated.

Relative abundances of identified phosphopeptides were estimated via comparison to known amounts of internal standards and adjusted for recovery of internal phosphostandards spiked in at various steps throughout the procedure. Copy per cell equivalents were calculated based on the assumption that one gram of tissue is equivalent to 1e9 cells and that 1.6 femtomoles of material is representative of one copy per cell.

### Downstream data analysis

The majority of the analysis was performed in Python (2.7.12 or 2.7.15), with the following major packages: scipy (1.1.0); numpy (1.15.3); matplotlib (2.2.3); pandas (0.23.4); seaborn (0.9.0); matplotlib_venn; upsetplot (0.1); acora (2.2), and modlamp (4.1.1).

Only peptides of length 8 to 15 amino acids inclusive were considered in this analysis. All modifications were stripped and duplicated sequences combined prior to downstream processing analysis. For standard (non-phospho-enriched) immunopeptidomes abundance values were ignored where present, due to the incompatibility of different scales and the lack of abundance values across all datasets.

The peptide-MHC binding prediction software MHCflurry (1.2.2)^17^ was used to assign likely HLA allele binding alleles and predict affinities, being called in Python instead of run standalone, with predicted affinity values under 1000 nM being assigned as a low-threshold potential binder. Peptides were assigned to the allele with the best predicted affinity, i.e. lowest nM value. NetMHC-4.0 was used^18^ to confirm MHCflurry findings, being run as a standalone tool with default parameters for the relevant HLA alleles, with a rank score of under 2 being assigned as a potential binder.

WebGestalt^38^ was run via the web interface using the following conditions: ‘hsapiens’, Overrepresentation Enrichment Analysis, geneontology, Biological_Process, collecting results with a false discovery rate (FDR) < 0.05. Gene symbols of proteins that had peptides featured in the immunopeptidomes of all samples of a given group (i.e. culture or xenograft grown cells) were compared against a reference list containing the gene symbols of all human proteins listed in SwissProt (accessed in November of 2018).

Overlap of mapped human peptides with the mouse proteome was accomplished by using the acora package in Python, which makes use of an Aho-Corasick string matching algorithm to rapidly search a sequence with a large panel of potential sub-strings^39^.

Electrophysical properties of peptides were calculated using the modlAMP Python package^40^. Charge values were calculated at pH 7.4, including C-terminal amides.

The Jaccard index is a measure of normalized overlap between two sets of elements, ranging from zero (meaning no overlap) to one (for comparison of sets with identical lists). It is defined as the intersection (number of shared elements) divided by the union (total number of unique elements across the two sets). The Jaccard of two lists of peptides A and B is calculated with the equation below (where ‘∩’ indicates peptides shared between both sets, and ‘∪’ indicates peptides present in the combination of the two sets). Note that this equation treats every unique peptide sequence as independent to every other, regardless of whether any two are similar. As peptides which differ by small changes (e.g. with single residue differences, or being shorter/longer versions of the same sequence) can be presented equivalently well this metric can be considered a conservative measure of immunopeptidome similarity.

$$J(A,B)=\frac{A\cap B}{A\cup B}$$

### Public data sources

JY RNAseq data was downloaded from NCBI via the SRA accession SRR364065^41^. Transcripts were quantified by pseudomapping using Kallisto^42^ (version 0.43.1) against the GRCh38 reference, supplemented with EBV ‘Akata’ strain transcripts^43^ (to prevent misassignment of viral sequences). JY proteome data came from a previous immunopeptidomic study by Bassani-Sternberg *et al*.^20^.

Previously published JY immunopeptidomes come from studies by Hassan *et al*.^16^, Bourdetsky *et al*.^44^, Bassani-Sternberg *et al*.^20^, and Caron *et al*.^45^. Other non-JY control immunopeptidomes come from the SysteMHC Atlas project^21^. Samples used were: MM16, a human melanoma sample^46^ (SYSMHC00023); THP1, a commonly used monocyte-like leukemia line, unpublished (SYSMHC00002); C1866, a T lymphoblastic line^47^ (SYSMHC00011). MDA-MB-436 and Colo205 were also used to generate immunopeptidomes as alternative cell lines to JY. HLA alleles of these lines as reported in SysteMHC/TRON are:

- MM16: HLA-A*01:01, HLA-A*24:02, HLA-B*07:02, HLA-B*08:01, HLA-C07:01, HLA-C*07:02
- THP1: HLA-A*02:01, HLA-A*24:02, HLA-B*15:11, HLA-B*35:01, HLA-C*03:03
- C1866: HLA-A*01:01, HLA-A*01:01, HLA-B*08:01, HLA-B*44:02, HLA-C*05:01, HLA-C*07:02
- MDA-MB-436: HLA-A*01:01, HLA-B*08:01, HLA-C*07:01
- Colo205: HLA-A*01:01, HLA-A*02:01, HLA-B*07:02, HLA-B*08:01, HLA-C*07:01

Note that MHCflurry version 1.2.2 does not support binding predictions for HLA-C*07:01: instead HLA-C*07:02 (which has a very similar binding motif) was used for predicting likely responsible alleles for Colo205 and MDA-MB-436.

Whole proteomes were obtained from Uniprot, with the accessions UP000005640 (human) and UP000000589 (mouse).

## Supplementary information

Supplementary Figures 1–13

## Data Availability

The data that support the findings of this study were generated in collaboration with a third party and certain restrictions apply to data availability. The non-enriched peptide data and accompanying code used to generate the plots in this paper are available for download from the repository from GitHub at https://github.com/JamieHeather/xenoimmunopeptidomics. Alternatively the exact release used here can be downloaded via Zenodo from 10.5281/zenodo.3554754. Due to third party data restriction policies, 48 phosphopeptide sequences have been redacted from these data. However the analysis produces the same results with or without these sequences.
