# Supplementary material for: Murine xenograft bioreactors for human immunopeptidome discovery
Source: Sci Rep. 2019 Dec 6;9:18558. doi: 10.1038/s41598-019-54700-2 (PMC6898210; doi:10.1038/s41598-019-54700-2)
Supplement: Supplementary file 1 — Supplementary Figures 1–13 [file 41598_2019_54700_MOESM1_ESM.pdf]

Murine xenograft bioreactors for human immunopeptidome discovery

Supplementary Figures 1-13

Authors:

James M. Heather[*1,2], Paisley T. Myers[3], Feng Shi[1,2], Mohammad Ovais Aziz-Zanjani[4], Keira E. Mahoney[4], Matthew Perez[3], Benjamin Morin[3], Christine Brittsan[3], Jeffrey Shabanowitz[4], Donald F. Hunt[4,5], and Mark Cobbold[*1,2].

[*] Corresponding authors

Affiliations:

1) Center for Cancer Immunology, Massachusetts General Hospital, Boston, Massachusetts, USA
2) Department of Medicine, Harvard Medical School, Boston, Massachusetts, USA
3) Agenus Inc., Lexington, Massachusetts, USA
4) Department of Chemistry, University of Virginia, Charlottesville, Virginia, USA
5) Department of Pathology, University of Virginia, Charlottesville, Virginia, USA

SFig 1: Input sample properties

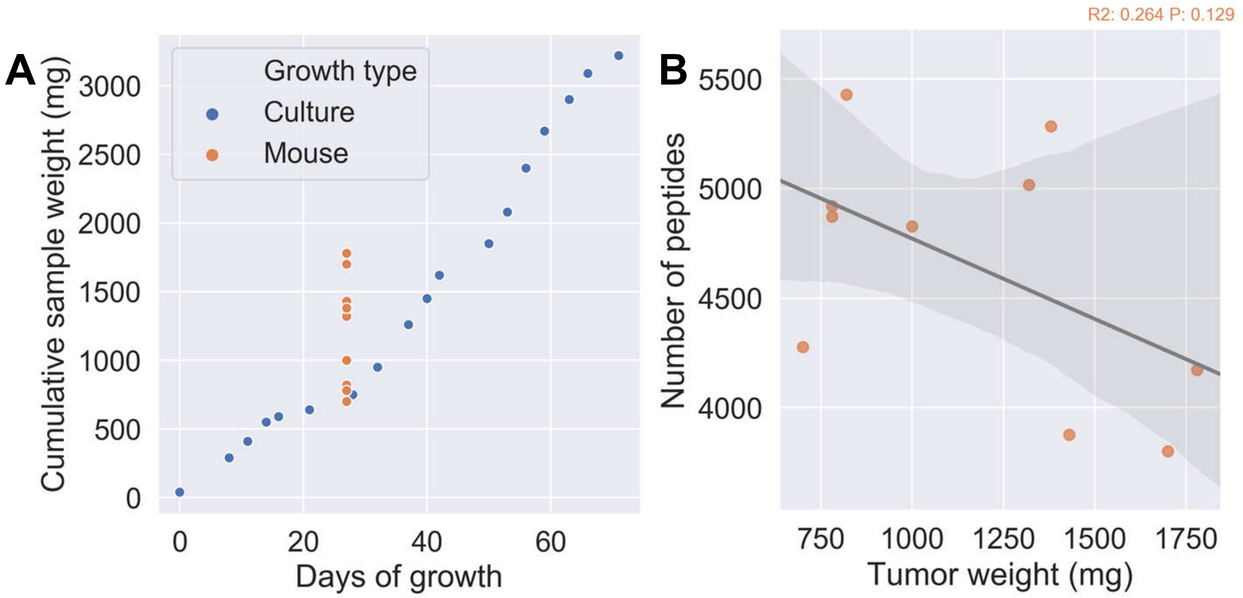

A: Cumulative weight over time of samples banked after serial cell culture collection (blue) or after implantation into NSG mice (orange).

B: Linear regression between the tumor weight achieved during the xenograft and the number of peptides produced from those samples, showing no correlation (statistics of the regression shown in the top right).

Sfig 2: Xenograft non-predicted binding peptides do not appear to be artefacts

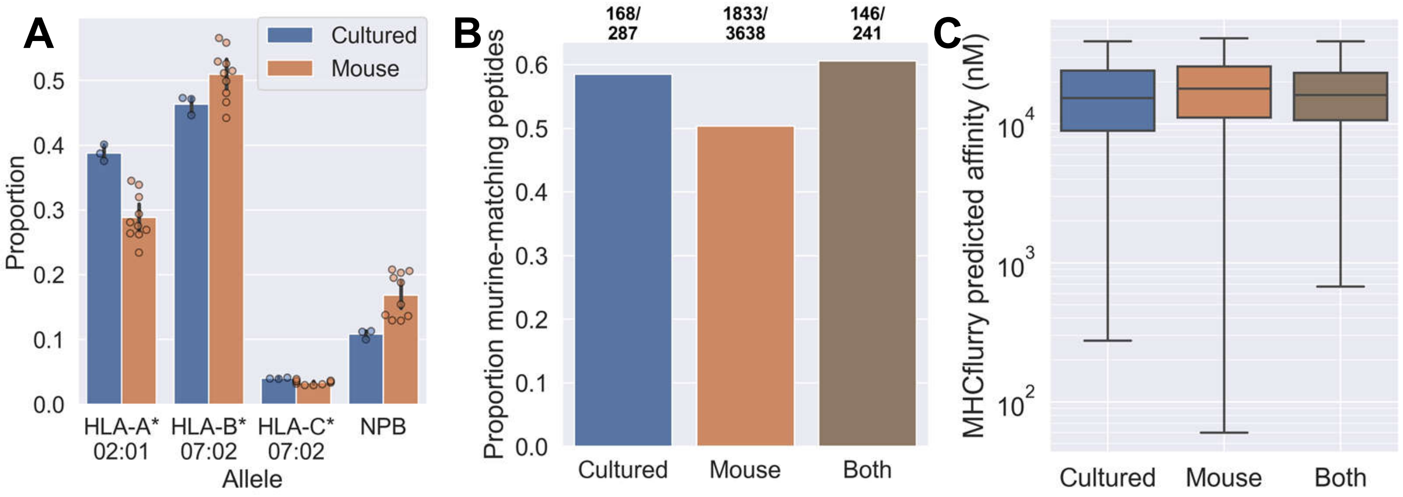

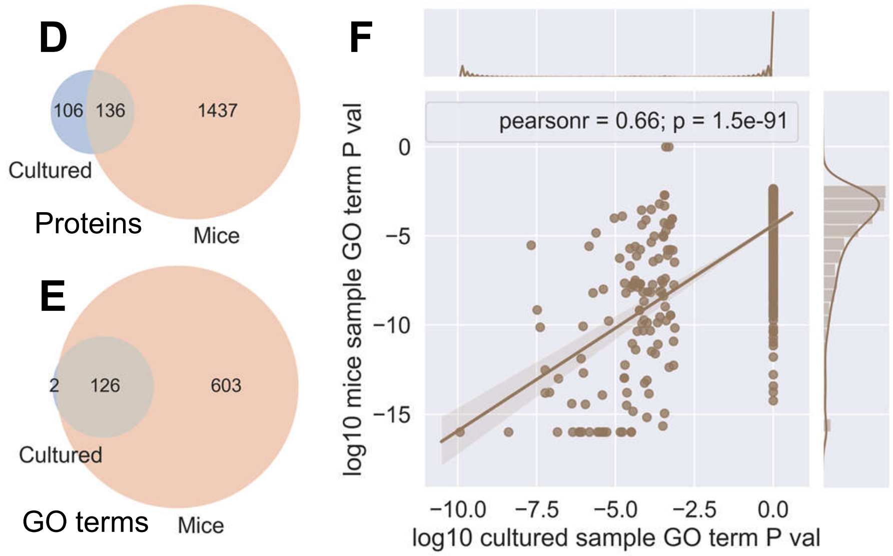

A: netMHC-4.0 predicted HLA binding contributions of JY immunopeptidomes of *in vitro* cultured versus murine xenograft grown cells. Default thresholds used; anything with a ranked score ≤ 2 (inclusive of both 'strong' and 'weak' binders) was counted as a binder, anything higher was deemed to have no predicted binder (NPB). Note that this threshold is not necessarily equivalent to the thresholds used for the MHCflurry predictions shown elsewhere in this paper.

B: Proportions of JY peptides (found exclusively in either cultured or xenograft cells, or both) which were predicted by MHCflurry to not bind to a relevant allele, whose sequence also appears in the reference mouse proteome (Uniprot accession UP000000589, accessed in November 2018). Boldened numbers above show the number of positive murine-proteome hits over the number of total non-predicted binding peptides in that group.

C: The same peptides as in **B** (those not predicted to bind to any JY HLA allele) went through another round of MHC prediction, now using the H2d murine haplotype alleles found in NSG mice: H-2Dd, H-2Kd, and H-2Ld.

D: Overlap in proteins represented by JY immunopeptides not predicted to bind any relevant MHC-I alleles, which were found exclusively either in cultured repeats (left, blue) or mouse-grown (right, orange) samples. Note that only peptides which could map to a single protein in the reference proteome were considered.

E: As in D, but showing overlap in enriched GO terms of proteins featured, determined by overrepresentation analysis via WebGestalt (FDR < 0.05).

F: Linear regression of the P value of GO term evalue of 0 were rounded down to the nearest order of magnitude ($1x10^{-16}$). Note that control experiments using length-matched random lists of proteins subsampled from the background list failed to yield any significant enriched GO terms.
nrichment of terms shown in E. GO terms not shared were assigned a P value of 1, while those with a

Sfig 3: Xenograft non-predicted binding peptides are more charged and more polar

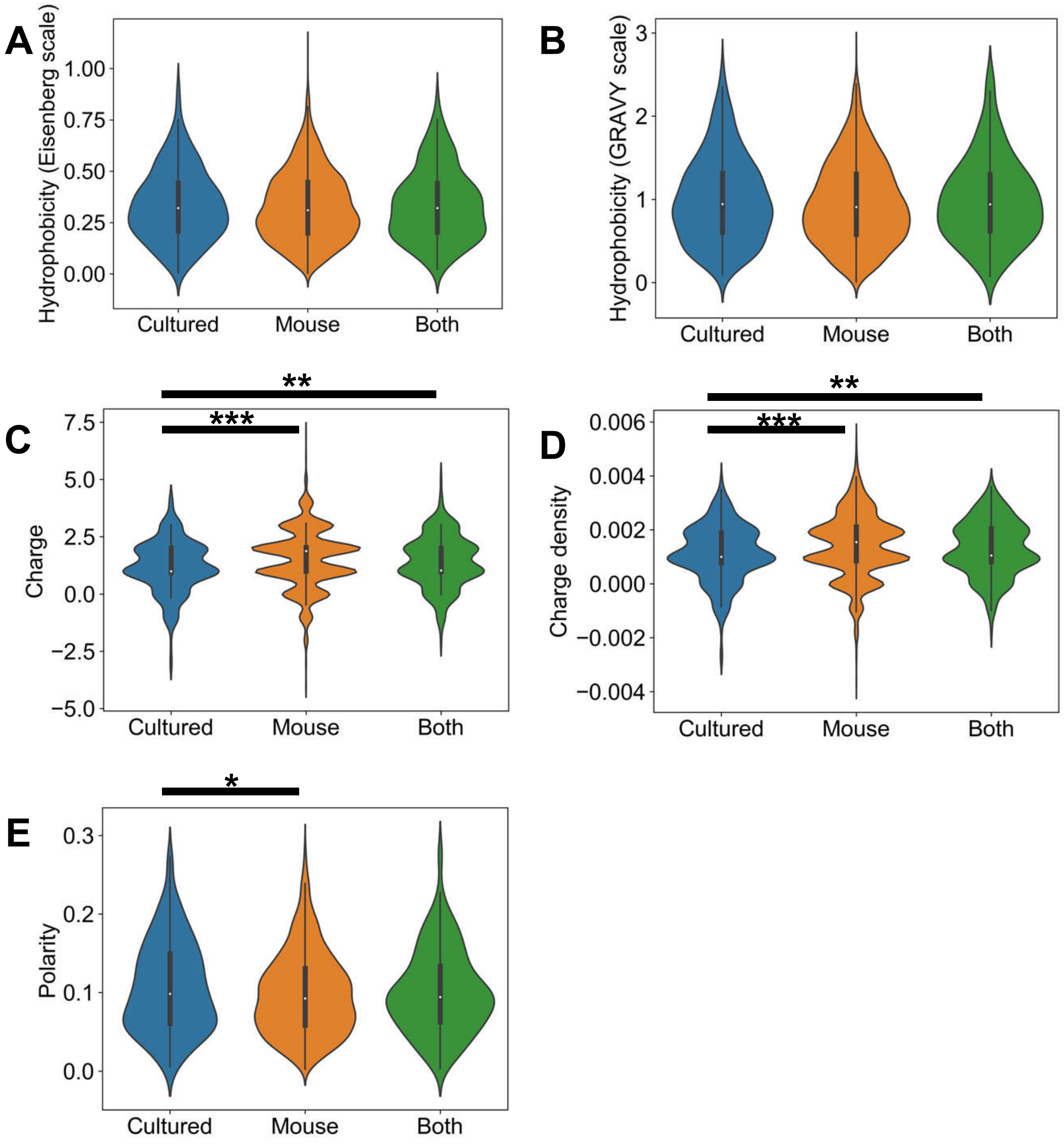

Investigation of the electrophysical properties of the peptides found in JY samples (solely in cultured or mouse repeats, or in both) which were not predicted to bind any of JY's HLA alleles. All values calculated using the modlAMP software package. The violinplots show two different scales of hydrophobicity (**A** & **B**, Eisenberg and GRAVY respectively), total molecular charge and per molecular weight charge density (**C** & **D** respectively), and total polarity (**E**, shown on the Zimmerman scale). Significant differences by Mann Whitney U test are shown (p < 0.05 = *; p < 0.01 = **; p < 0.001 = ***).

SFig 4: Gibbs cluster results for all immunopeptidome samples

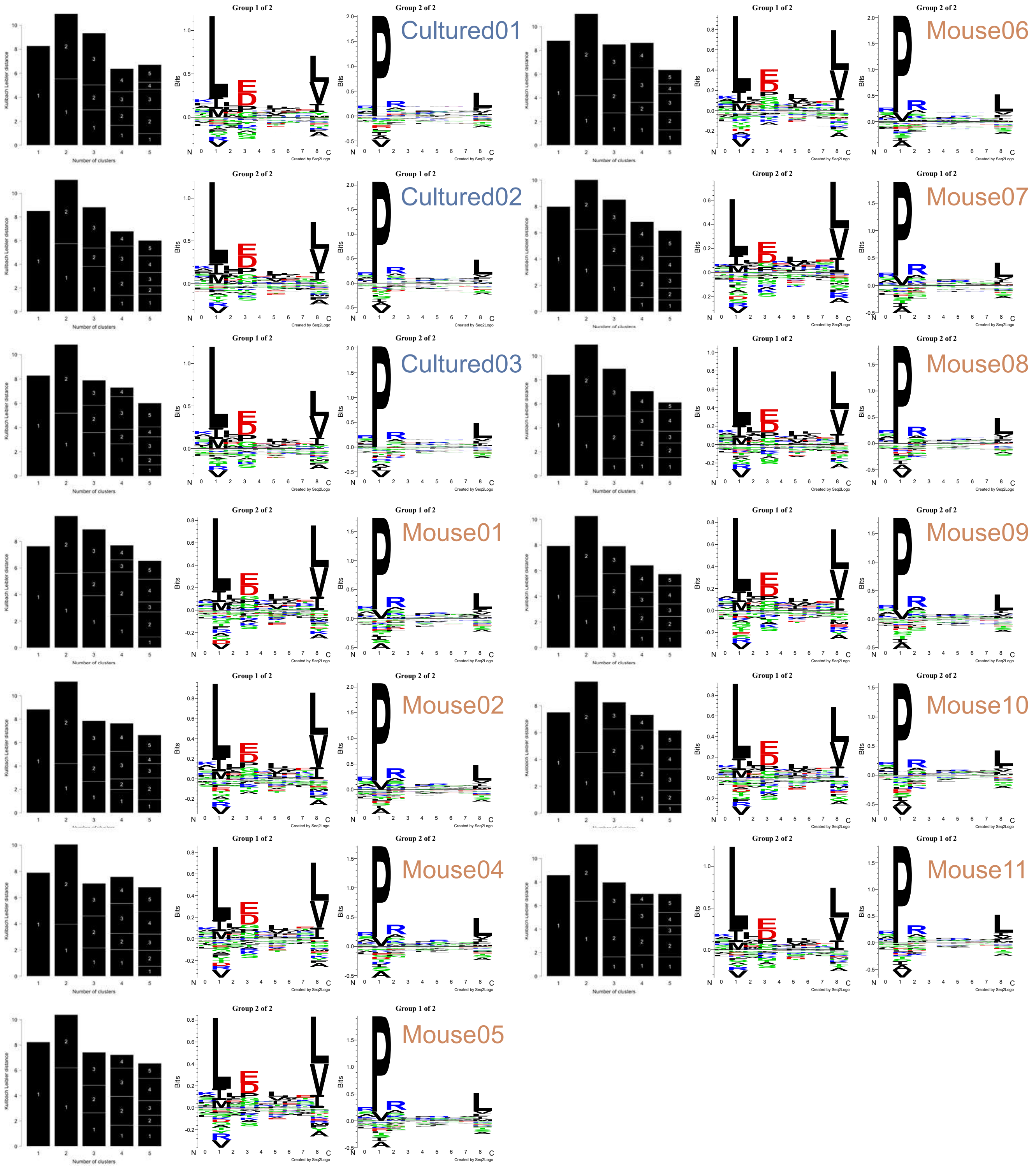

GibbsCluster-2 motifs for all of the JY immunopeptidome samples produced in this study, using default program settings. Results shown are for two clusters, as this value had the highest Kullback Leibler distances across all samples.

SFig 5: Correlation of protein representation in the immunopeptidome with published JY transcriptome data

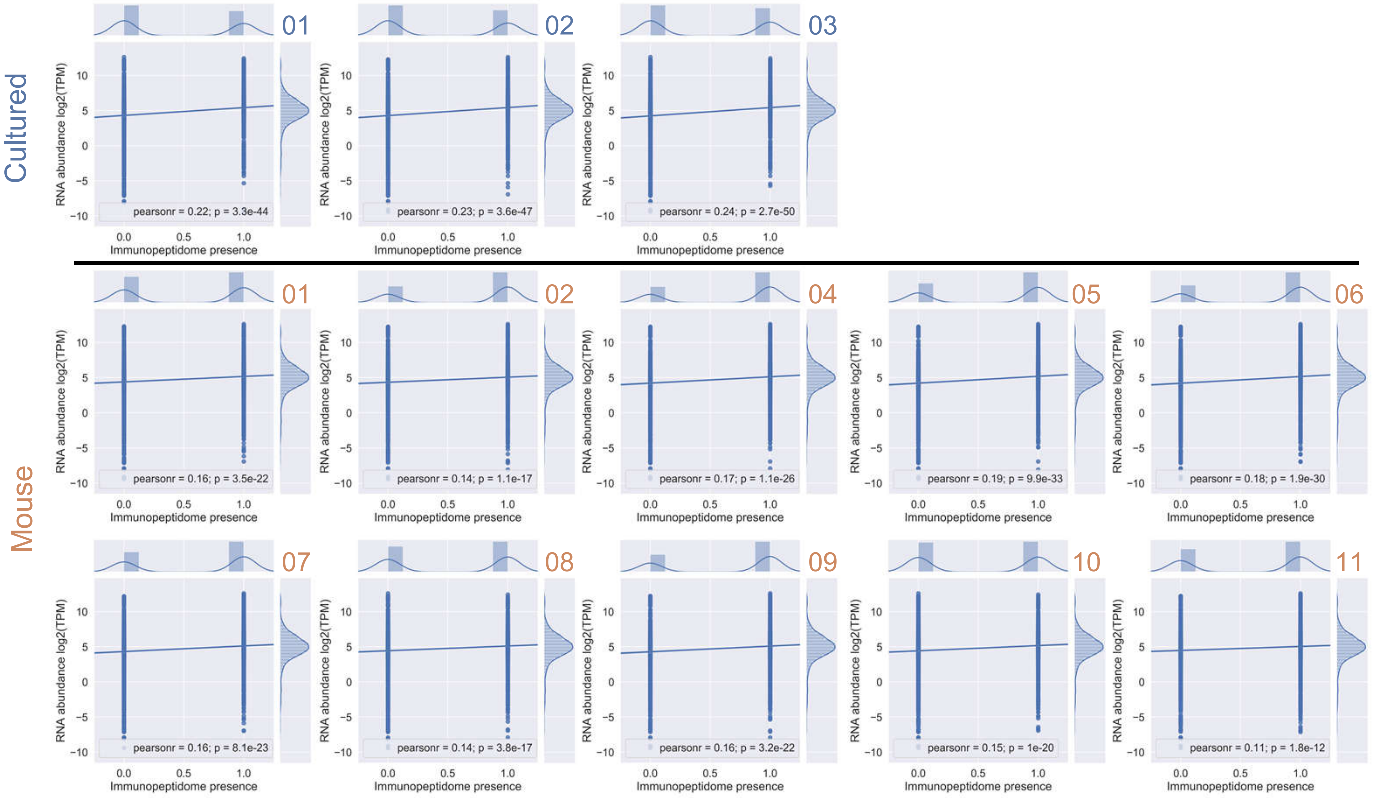

Correlation of the binarized presence/absence of proteins featured in JY immunopeptidomes with the abundance of that protein’s pooled transcripts (from a previously published JY RNAseq dataset). Marginal histograms show the distribution of data along each axis.

SFig 6: Correlation of protein representation in the immunopeptidome with published JY proteome data

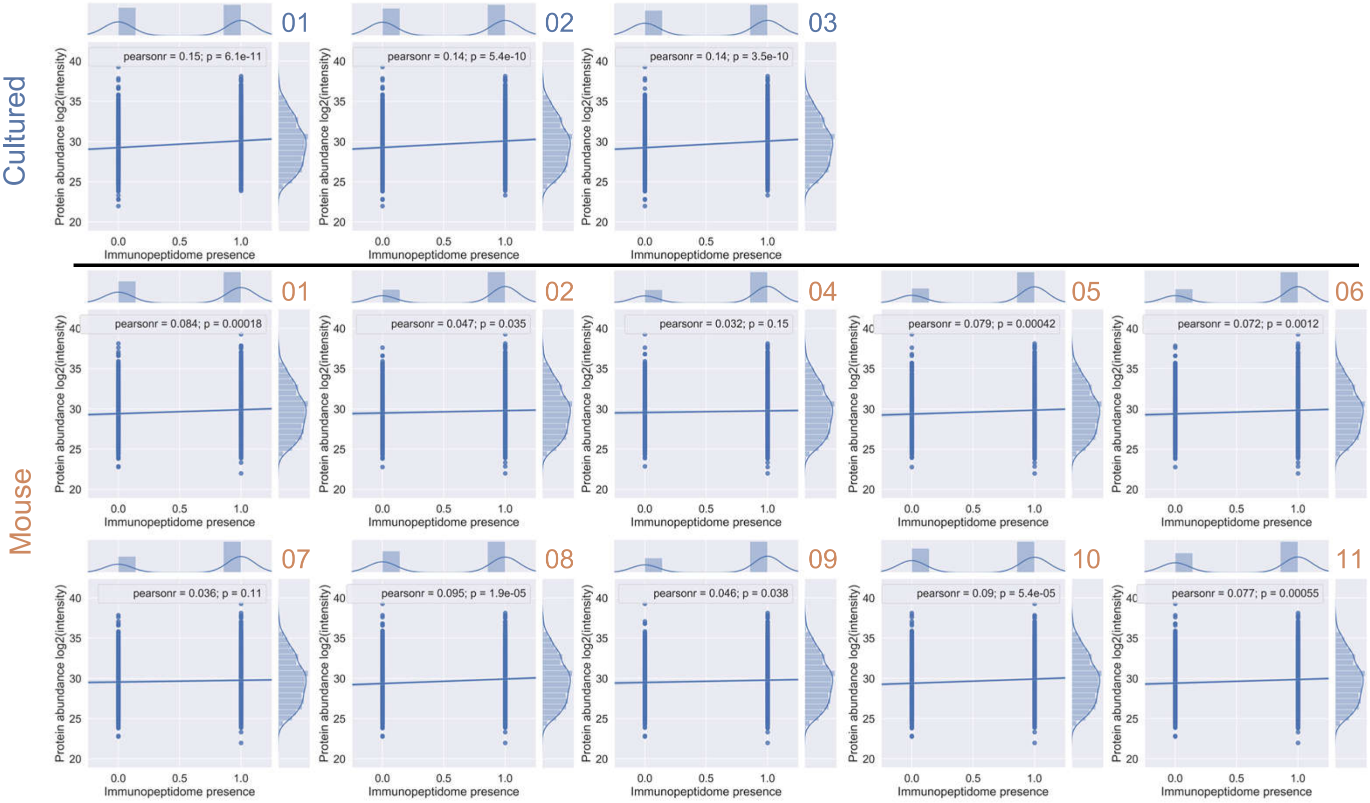

Correlation of the binarized presence/absence of proteins featured in JY immunopeptidomes with the abundance of that protein in JY (from a previously published JY proteomic dataset). Marginal histograms show the distribution of data along each axis.

SFig 7: WebGestalt over-representation analysis GO SLIM summaries

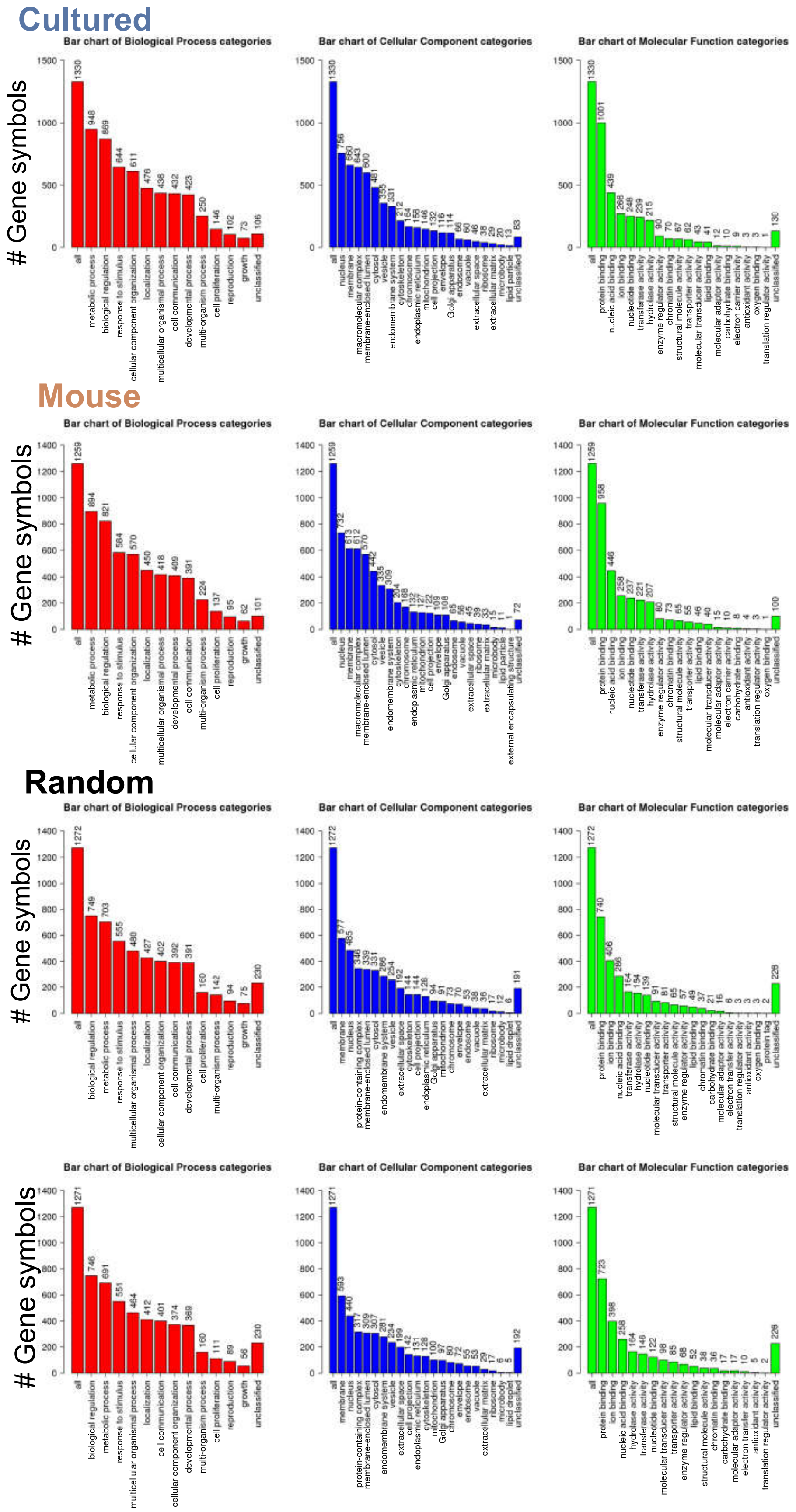

WebGestalt produced GO Slim summaries of proteins featured in the immunopeptidomes of JY cells grown in culture (top row) or as xenografts in mice (second row down). Only counting proteins with peptides featured in every sample of the specific growth type. Each analysis shows the number of gene symbols mapping to different properties in the ‘biological process’, ‘cellular component’ and ‘molecular function’ ontologies. Bottom two rows indicate two random samples of a similar number (1300) of gene symbols from the background list of all Uniprot proteins, so as to indicate expected distribution of random proteins.

SFig 8: Violinplot of grouped pairwise comparisons of subsampled immunopeptidomes

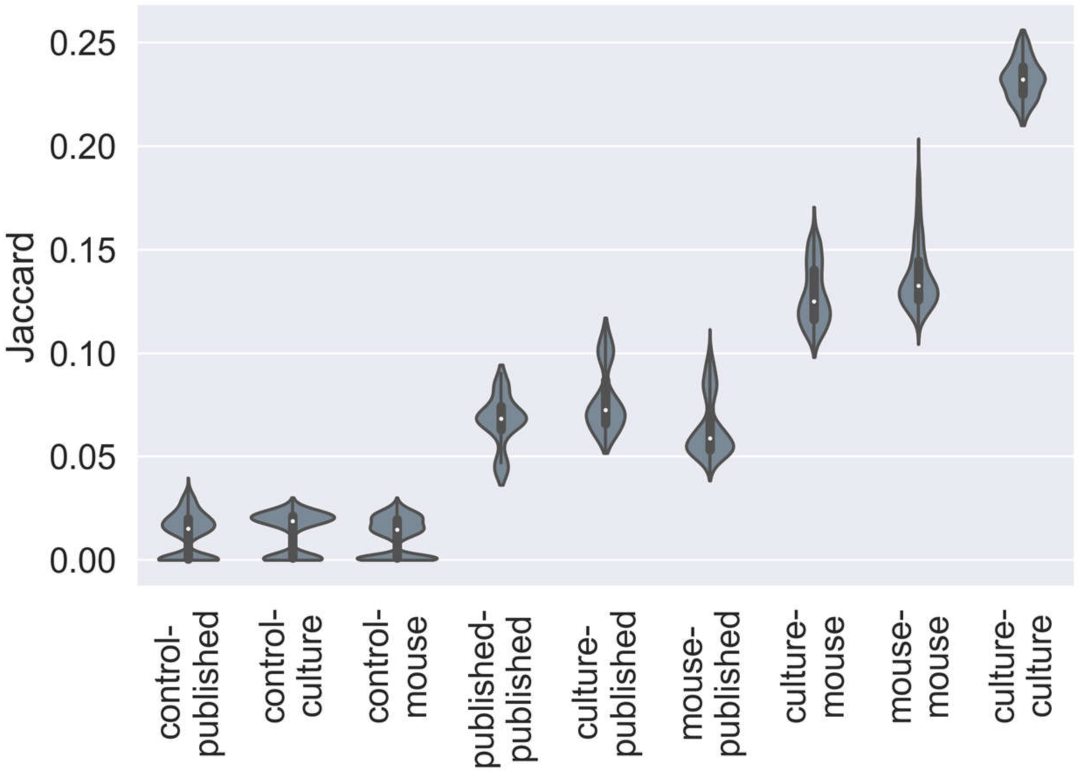

Violin plot of average Jaccard indexes produced from every different unique pairwise comparison of subsampled immunopeptidomes featured in Figure 4. 1500 peptides were randomly selected without replacement from each peptide file for Jaccard index calculated across 100 iterations, then averaged.

SFig 9: Individual pairwise overlaps between phospho-immunopeptidomes (with/without subsampling)

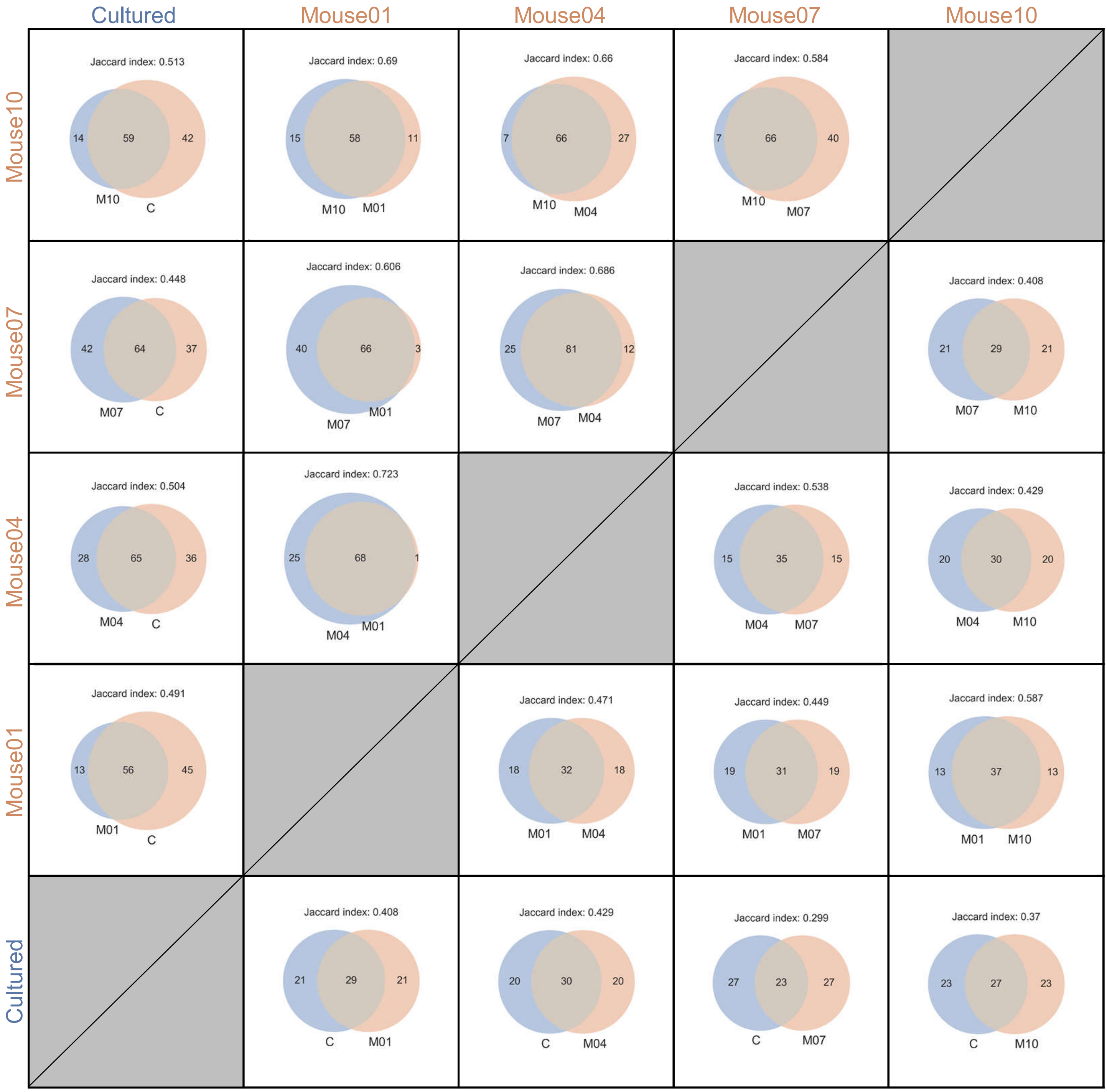

Venn diagrams and Jaccard indexes of the individual pairwise comparisons of JY sample phosphopeptide sequences, of both whole lists (top left triangle) and subsampled lists (bottom right triangle). 50 sequences were randomly subsampled without replacement; just a single iteration is shown here. See Figure 5D for averaged values across 100 iterations.

SFig 10: Individual pairwise correlation of phosphopeptide abundance between samples

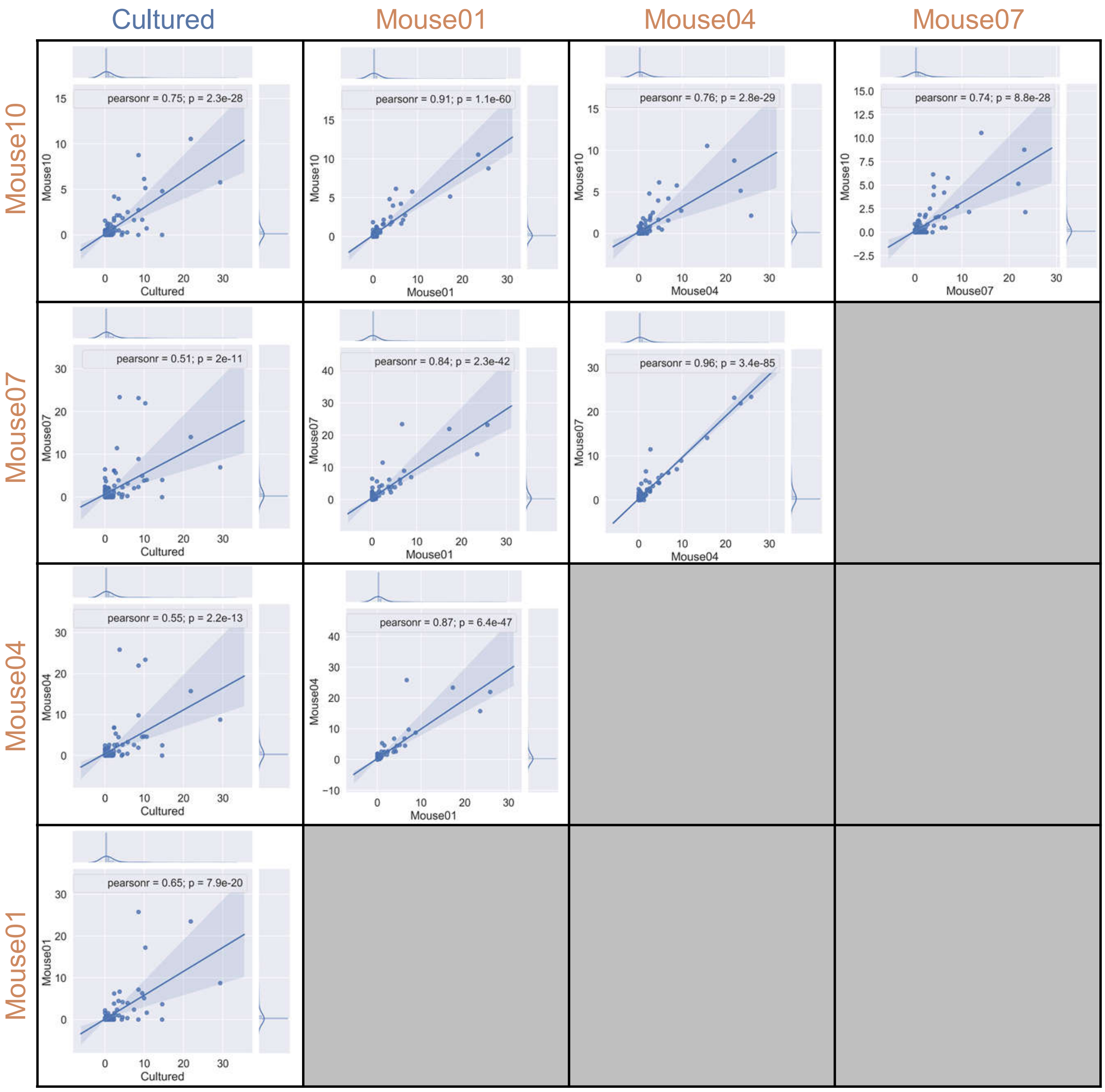

Plotting the correlation of phosphopeptide abundance between each unique pairwise combination. Both axes show (estimated) copies per cell. Marginal histograms show distribution across each axis. Overlain statistics indicate Pearsons R squared and p values of the fit. Shaded area shows bootstrapped 95% confidence intervals.

SFig 11: UpSet analysis of phosphopeptides

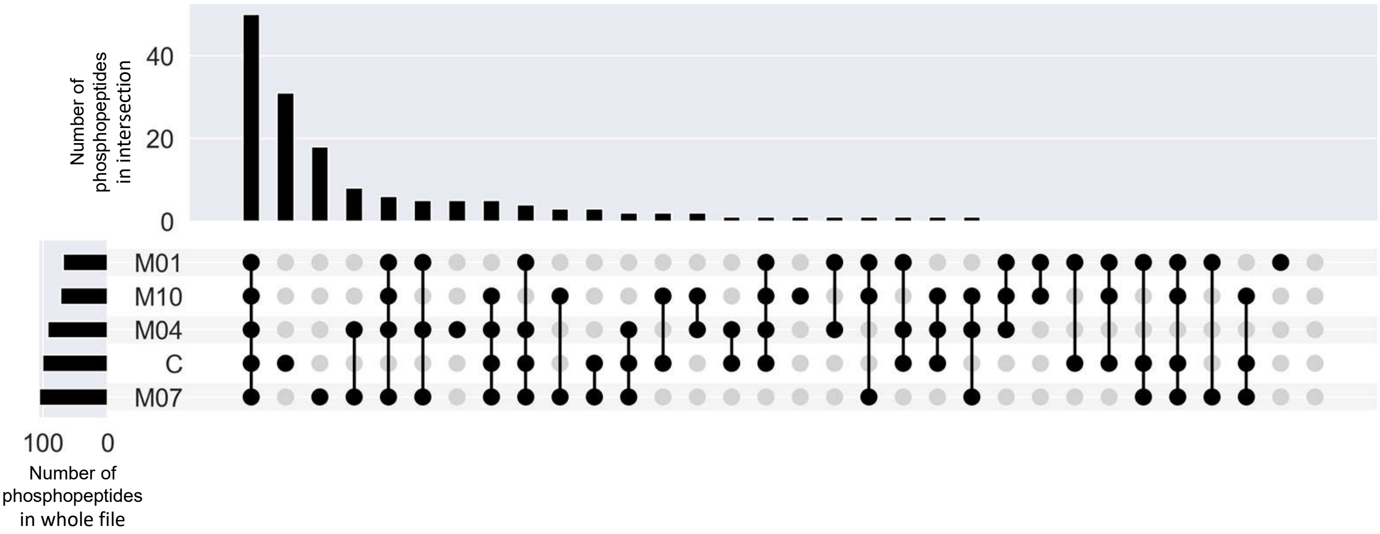

UpSet plot analysis showing distribution of phosphopeptide sequences across the various processed JY samples ('C' = cultured, 'M' = mouse xenograft). Dots along x axis indicate which samples define the set of the column above. I.e.: the first column indicates the number of peptides that area shared by all samples; the second column indicates those only found in the C sample; the third column indicates those only found in the M07 sample; the fourth column indicates those found only in both M04 and M07, and so on. Ordered by cardinality (i.e. the number of elements per set).

SFig 12: All phospho-immunopeptidome GibbsCluster-2 results

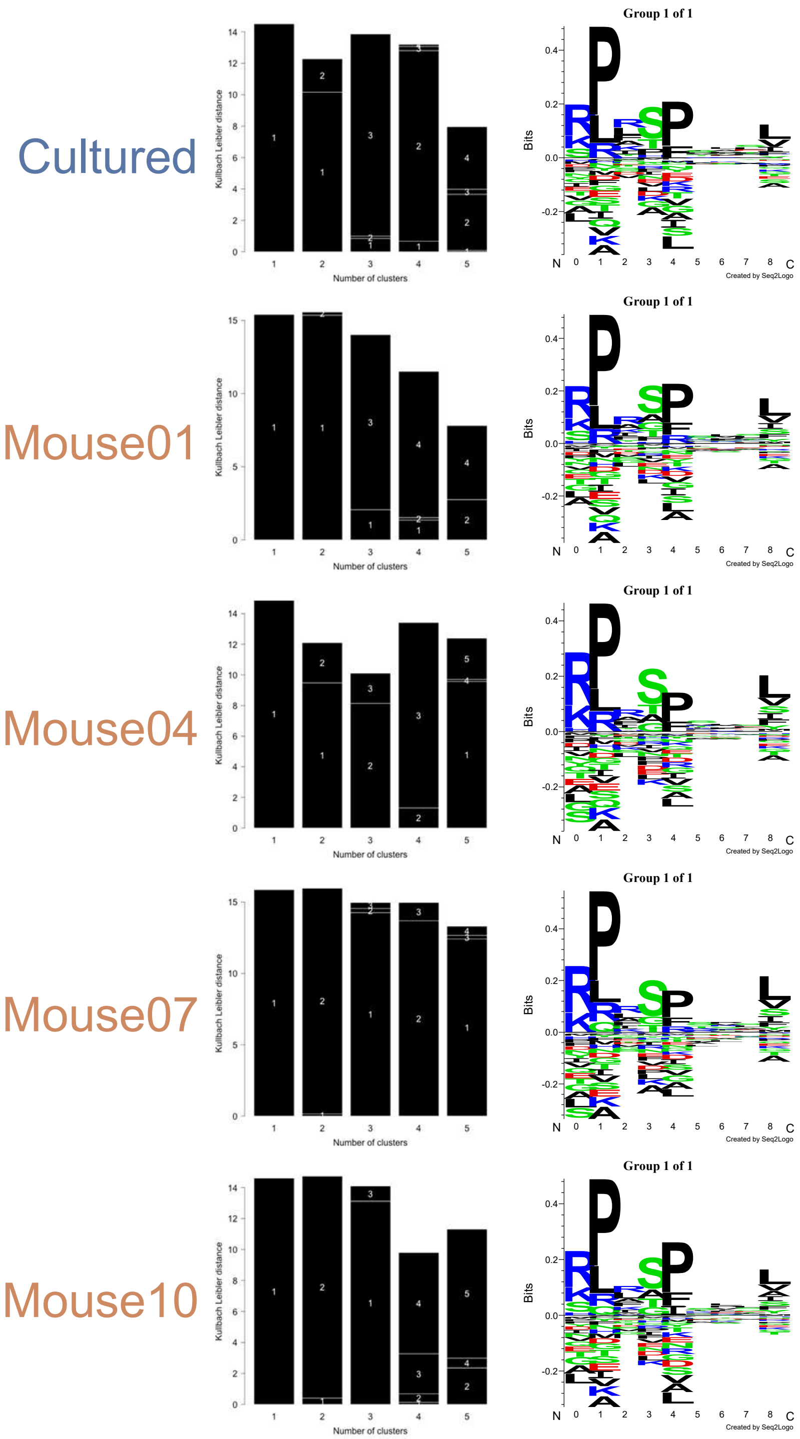

GibbsCluster-2 motifs for all of the JY phospho-immunopeptidome samples produced in this study, using default program settings. Results shown are for a single cluster, as this value had the highest Kullback Leibler distances across many samples (and where two clusters had a higher value the majority of the KLD was contributed to from a single cluster).

SFig 13: Comparison of *in vitro* vs *in muridae* growth for immunopeptidomics for two additional cell lines

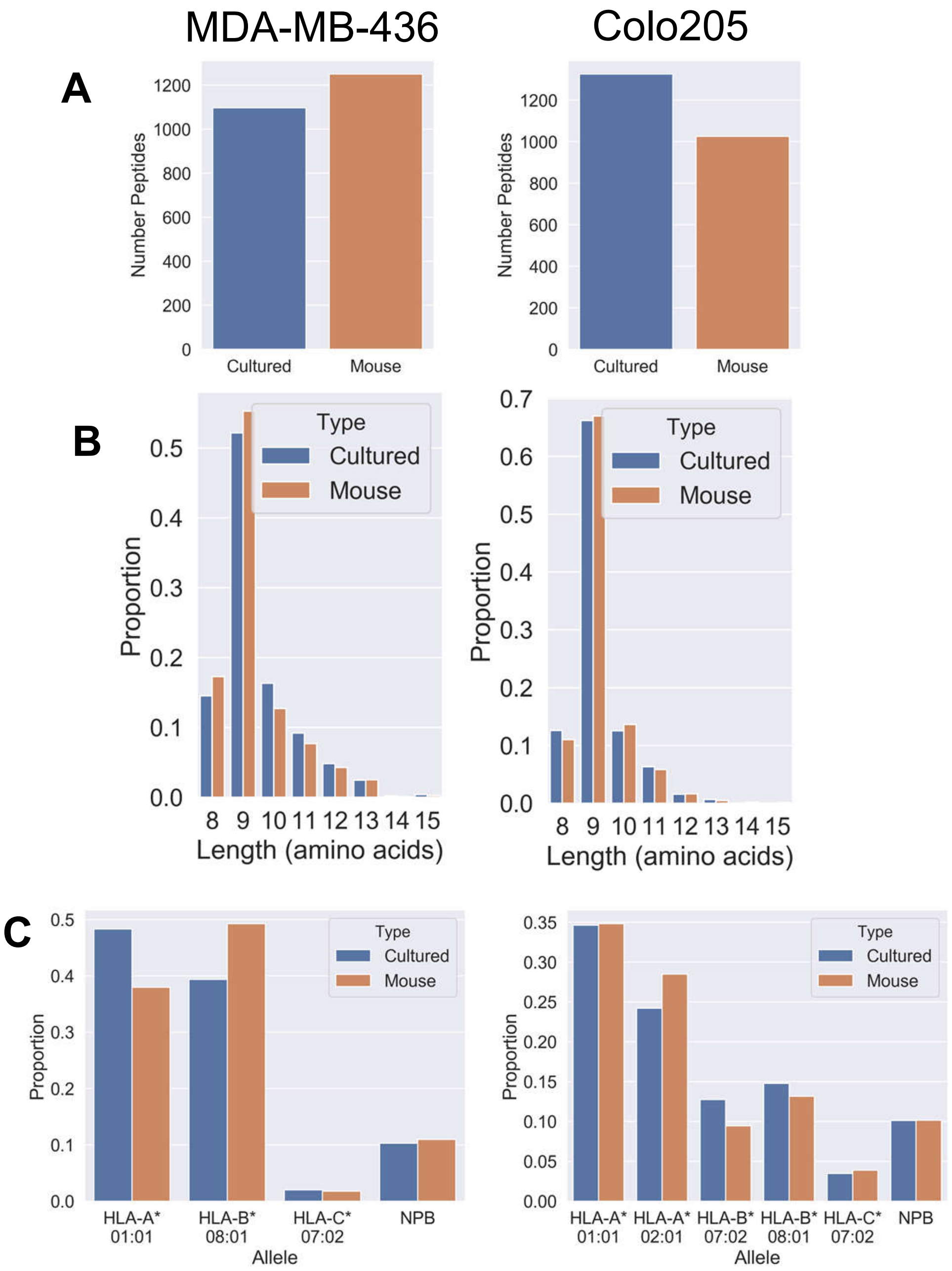

A: Number of peptides per immunopeptidome file for MDA-MB-436 cells (left) and Colo205 cells (right), grown either in culture (blue bars) or in mice (orange bars).

B: Proportion of peptides of each length (in the range 8 to 15 amino acids) for MDA-MB-436 (left) and Colo205 (right) cells.

C: Proportion of peptides predicted by MHCflurry to bind each of the relevant HLA alleles for the two cell additional cell lines.
